# Unique Transcriptome Patterns of the White and Grey Matter Corroborate Structural and Functional Heterogeneity in the Human Frontal Lobe

**DOI:** 10.1371/journal.pone.0078480

**Published:** 2013-10-23

**Authors:** James D. Mills, Tomas Kavanagh, Woojin S. Kim, Bei Jun Chen, Yoshihiro Kawahara, Glenda M. Halliday, Michael Janitz

**Affiliations:** 1 School of Biotechnology and Biomolecular Sciences, University of New South Wales, Sydney, New South Wales, Australia; 2 Neuroscience Research Australia, Sydney, New South Wales, Australia; 3 School of Medical Sciences, University of New South Wales, Sydney, New South Wales, Australia; 4 National Institute of Agrobiological Sciences, Agrogenomics Research Center, Bioinformatics Research Unit, Tsukuba, Ibaraki, Japan; The John Curtin School of Medical Research, Australia

## Abstract

The human frontal lobe has undergone accelerated evolution, leading to the development of unique human features such as language and self-reflection. Cortical grey matter and underlying white matter reflect distinct cellular compositions in the frontal lobe. Surprisingly little is known about the transcriptomal landscape of these distinct regions. Here, for the first time, we report a detailed transcriptomal profile of the frontal grey (GM) and white matter (WM) with resolution to alternatively spliced isoforms obtained using the RNA-Seq approach. We observed more vigorous transcriptome activity in GM compared to WM, presumably because of the presence of cellular bodies of neurons in the GM and RNA associated with the nucleus and perinuclear space. Among the top differentially expressed genes, we also identified a number of long intergenic non-coding RNAs (lincRNAs), specifically expressed in white matter, such as LINC00162. Furthermore, along with confirmation of expression of known markers for neurons and oligodendrocytes, we identified a number of genes and splicing isoforms that are exclusively expressed in GM or WM with examples of *GABRB2* and *PAK2* transcripts, respectively. Pathway analysis identified distinct physiological and biochemical processes specific to grey and white matter samples with a prevalence of synaptic processes in GM and myelination regulation and axonogenesis in the WM. Our study also revealed that expression of many genes, for example, the *GPR123*, is characterized by isoform switching, depending in which structure the gene is expressed. Our report clearly shows that GM and WM have perhaps surprisingly divergent transcriptome profiles, reflecting distinct roles in brain physiology. Further, this study provides the first reference data set for a normal human frontal lobe, which will be useful in comparative transcriptome studies of cerebral disorders, in particular, neurodegenerative diseases.

## Introduction

The human cerebrum is extraordinarily complex and is composed of billions of neurons and trillions of synaptic connections. Neurons are organized into circuit assemblies that are modulated by specific interneurons and non-neuronal cells. The frontal lobe is often considered the most highly developed and most human featured brain region. As the prefrontal and frontal cortexes exert executive type control over other structures, they are expected to show significant connectivity to other brain regions. This part of the brain manages the most complex thought, decision making, planning, conceptualization, attention control, and working memory [1,2].

Broadly speaking, the human cortex can be divided into the phylogenetically older allocortex and the newer neocortex. The neocortex has expanded the most in humans and is composed of six superimposed layers with distinct cellular compositions; the thickness of each layer differs in each cortical lobe [3]. The most prominent distinction of the cerebrum is its division into outer cortical grey matter (GM) and inner white matter (WM). GM consists of neural cell bodies, their dendrites, and parts of their axons, as well as glial cells, mainly astrocytes. In contrast, WM is mainly aggregations of myelinated and non-myelinated axons linking different cortical and subcortical regions [4]. Of long-neglected significance, WM has recently started to be a subject of intensive studies due its involvement in the development of working memory capacity and reading ability [5]. Recently, the frontal WM has been proposed as a major contributor to human brain enlargement and higher structural connectivity, as compared to other primates [6,7].

Despite several RNA-Seq-based studies investigating whole transcriptome profiles of cerebral tissue in pathological conditions such as Alzheimer’s disease (AD) [8,9] or autism [10] there has been no systematic attempt to investigate transcriptomic landscape of normal brain tissue with resolution of RNA sequencing. Hawrylycz and colleagues recently published a microarray-based transcriptome profile of the distinct brain regions in two individuals [11]. They provided important insights into the spatial distribution of expression across well-defined neuroanatomical regions. The critical conclusion of this study was the importance of local gene expression patterns for the maintenance of physiological uniqueness within these regions. Because this analysis was performed using microarray technology, it does not provide further information on posttranscriptional control in the brain, namely, alternative splicing.

Here, we report, for the first time, detailed transcriptome profiles of GM and WM of the human lobe using RNA-Seq. Comparative analysis of the gene and isoform expression, combined with the pathway analysis, revealed surprisingly distinct transcriptome patterns reflecting the contribution of different glial cell types and neuronal structures to WM and GM, respectively. Moreover we observe elevated expression of lincRNAs in WM, as well as isoform switching between WM and GM for genes encoding DNA binding proteins and proteins involved in signal transduction.

## Materials and Methods

### Human brain tissue

Human brain tissues were obtained from the Sydney Brain Bank and NSW Tissue Resource Centre, part of The Australian Brain Bank Network funded by the National Health and Medical Research Council of Australia. Ethics approval was from the University of New South Wales Human Research Ethics Committee. Frozen brain tissue samples from superior frontal gyrus (SFG) GM and WM were collected from three individuals aged 79, 94 and 98. The PMI of samples ranged 8-24 hrs and pH 5.77-6.65. All three brains were pathologically diagnosed and were free of any pathology or neurodegeneration.

### RNA isolation, library preparation and sequencing

Total RNA was isolated using RNeasy Lipid Tissue Midi Kit (Qiagen) followed by RNase-free DNase treatment to remove traces of genomic DNA. The RNA quality of the total RNA was assessed using the Agilent 2100 Bioanalyser RNA Nano Chip and the RIN values ranged between 6.0 and 7.0. This RIN range was previously shown to have a little effect on relative gene expression ratios [12] (and our unpublished observations). Six RNA samples (three WM and three GM) were prepared for sequencing according to the Illumina TruSeq RNA sample preparation guide and subjected to 100 bp paired-end sequencing using Illumina HiSeq1000. The sequence data have been submitted to the NCBI Short Read Archive with accession number SRA091951.

### Mapping of RNA-Seq reads using TopHat

Bioinformatics analysis was carried out using Galaxy; an open access web-based program that contains a variety of next-generation sequencing analysis tools including, TopHat and the Cufflinks package [13-15]. The Galaxy server was based at the Garvan Institute, Sydney, Australia. Using TopHat the reads were processed and aligned to the *H. sapiens* reference genome (build hg19). TopHat utilizes the ultra high-throughput short read aligner Bowtie to align the RNA-Seq reads, the reads are then analyzed and splice junctions between the exons are identified [16].The default parameters for TopHat were used. Subsequently the aligned reads from each sample were analyzed for 5’-3’ end bias using RSeQC [17]. 

### Transcript assembly with Cufflinks

The aligned reads were processed with Cufflinks. Cufflinks assembles the RNA-Seq reads into individual transcripts, inferring the splicing structure of the genes [18]. Cufflinks assembles the data parsimoniously giving a minimal set of transcripts that fits the data. Cufflinks normalizes the RNA-Seq fragment counts to estimate the abundance of each transcript. Abundance was measured in the units of fragments per kilobase of exon per million fragments mapped (FPKM). For this analysis a .GTF annotation file (iGenomes UCSC hg19 gene annotation) was used to guide the assembly. 

### Differential analysis with Cuffmerge and Cuffdiff

The alignment files produced by TopHat are merged for CuffDiff processing so that combinatorial pairwise sample comparison is performed. The output GTF files from each of the Cufflinks analysis and the .GTF annotation file were sent to Cuffmerge [18]. Cuffmerge takes these files and amalgamates them into a single unified transcript catalog; it also filters out any transcribed fragments that may be artifacts. The inclusion of the reference annotation allows gene names and other details such as, transcript ID, exon number, transcription start site ID and coding sequence ID to be added to the merged transcript catalogue. It also allows for the gene and transcripts to be classified as known or novel. The merged GTF file was then fed to Cuffdiff along with the original alignment files produced from TopHat. Cuffdiff takes the replicates from each condition and looks for statistically significant changes in gene expression, transcript expression, splicing and promoter use. Cuffdiff uses a corrected p-value, known as the q-value to determine if the differences between the two groups are significant (q-value<0.05).

### Visualization with CummeRbund and Interactive Genome Viewer

The resultant Cuffdiff output files were fed into CummeRbund. CummeRbund is an R package that is designed to simplify the analysis of the Cuffdiff outputs. CummeRbund is user friendly and allows for easy data exploration and figure generation [19]. The Broad Institutes Integrative Genome Viewer (IGV) (http://www.broadinstitute.org/igv/), was used to visualize Cufflinks GTF outputs, this allowed for comparisons to be made between genes of known structure and the gene structure of novel transcripts identified by Cufflinks [20,21].

### Gene-set enrichment analysis with DAVID

The gene list of differential expressed genes was split into two groups; those up-regulated in GM and those up-regulated in WM. Only annotated genes can be utilized by enrichment tools, all novel genes and indecisively annotated genes were removed. Each of these lists was fed into the Database for Annotation, Visualization and Integrated Discovery (DAVID) (http://david.abcc.ncifcrf.gov/) [22]. DAVID tested the gene ontology (GO) terms for over representation in each of the gene lists. The GO terms list produced by DAVID, were processed using the ‘Enrichment Map’ plug in for Cytoscape (http://www.cytoscape.org/) [23]. This produces a visual output of the text based GO term lists.

### In situ hybridization validation

Our RNA-Seq expression data were compared with *in situ* hybridization (ISH) data from the Allen brain atlas database (http://www.brain-map.org/), which uses RNA probes to measure gene expression in normal human dorsolateral frontal cortex.

## Results

### Total transcription in GM and WM

An expression catalogue containing 32,740 genes was created by the Cufflinks package. Of this catalogue, 18,362 of the genes were considered expressed in the analyzed brain samples. This number includes 3,615 unannotated genes, 44 small nucleolar RNAs (snoRNAs), 67 micro RNAs (miRNAs), 40 lincRNAs and 52 RNAs of uncharacterized function (locRNAs). Short non-coding RNA sequences were presumably derived from their polyadenylated long non-coding RNA parent transcript or might be carryovers captured during library preparation. The cumulative coverage of the expressed transcripts in GM and WM was approximately 37% of the entire human genome; this figure includes the transcribed introns that were spliced out. There was no significant difference between the total human genome coverage of GM and WM.

The developed isoform catalogue assembled by Cufflinks contained over 85,000 distinct isoforms (splice variants). Among these isoforms, 36,306 were identified as being expressed in the selected regions of brain tissue, including 3844 unannotated isoforms, which equates to approximately two isoforms per gene. The actual distribution of isoforms per gene reveals a different picture; there were 9,427 genes with 1 isoform and 8,653 genes with between two and seven isoforms. There were only 107 genes with eight or more isoforms. This suggests that almost 50% of genes undergo alternative splicing.

Analysis using RSeQC showed that the aligned reads from each RNA sample had a similar degree of bias towards the 3’ end of each transcript (Figure S1). This result was expected due to the poly-A selection of RNA for sequencing. Of note, the Cufflinks package, utilized in this study, has been designed to correct for any sequence bias that occurs due to steps undertaken during template preparation [24]. 

### Differentially expressed genes and isoforms

**Figure 1:**
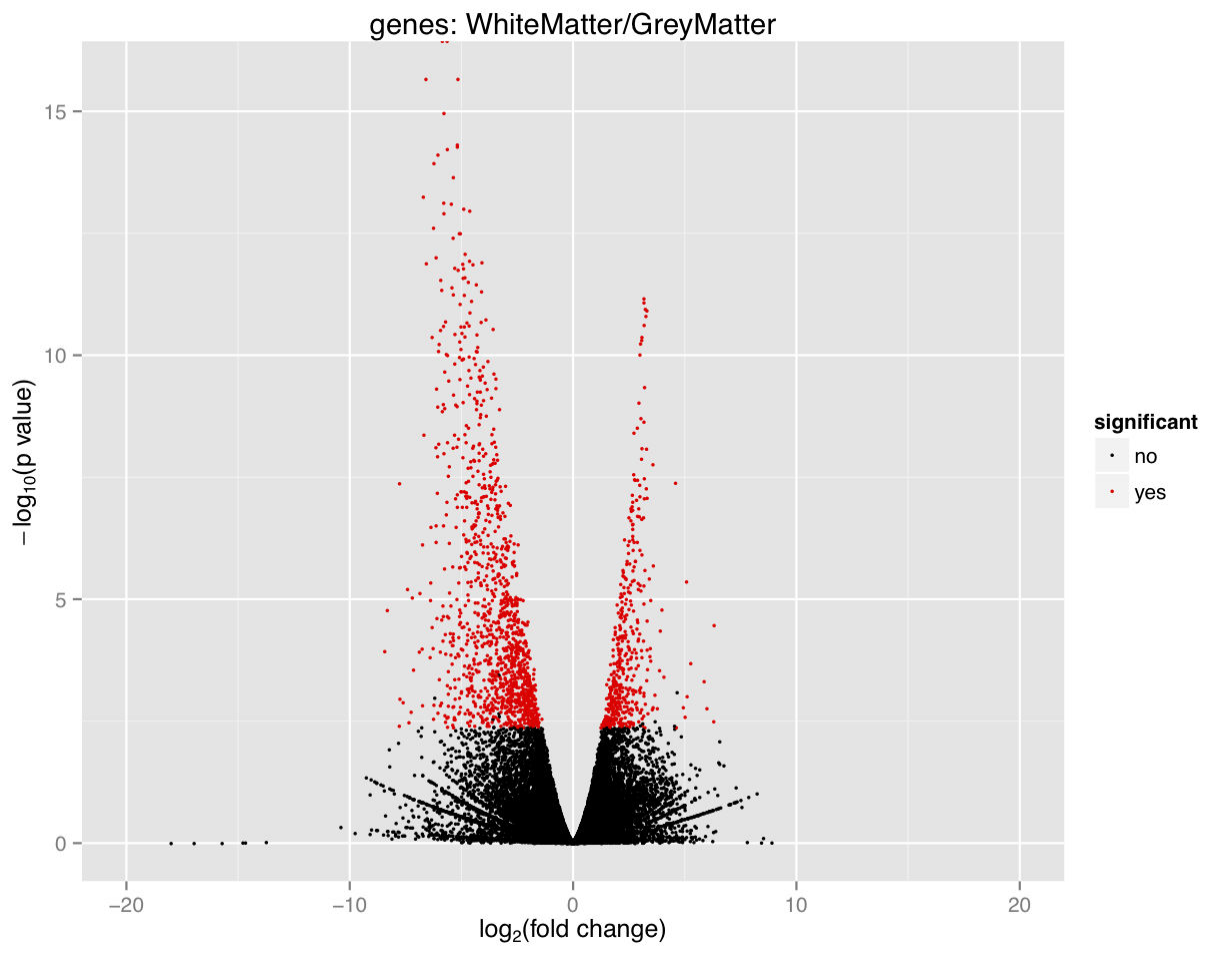
Volcano plot of gene expression in GM and WM. The fold-change of the genes was relative to their expression in WM. Those genes with a negative fold-change were up-regulated in GM (down-regulated in WM) and those genes with a positive fold-change were up-regulated in WM (down-regulated in GM). Genes that were statistically significant (q-value<0.05) are shown in red and were listed in Table S1. This figure demonstrates that a larger number of genes were significantly up-regulated in GM (1218) than were up-regulated in WM (434). Overall there was a greater spread of data for the genes that are up-regulated in GM.

Within in the set of 18,362 expressed genes, a total of 1,652 were identified as differentially expressed between the two conditions (q-value<0.05) (Figure 1). This included 1,218 that were up-regulated in GM and 434 that were up-regulated in WM (Table S1). Overall, there was a transcription bias toward GM. To preclude possible discordant results the gene lists were filtered so that any gene with an FPKM<1 for both conditions was excluded. An FPKM<1 means that there is less than one fragment per million aligned fragments mapped onto a 1-kb exon; this can be considered the result of background noise arising from erroneous sequencing or statistical errors during mapping. This reduced the overall list of differentially expressed genes to 1,591, of which 1,162 genes were up-regulated in GM and 429 genes were up-regulated in WM, the respective top 10 up-regulated genes in GM and WM by fold-change are shown in Table 1. Of the top 10 up-regulated genes in GM, there were two unannotated genes, and among the top 10 up-regulated genes in WM, there were three unannotated genes. On further inspection of the size and location of these genes, it was established that these genes fit the criteria for lincRNAs. Further, there was one annotated lincRNA (*LINC00162*) in the top 10 up-regulated genes in WM. 

**Table 1:** Top 10 up-regulated genes in GM and WM.

**Up-regulated in GM**									
**Gene**	**Description**			**Chrom.**	**FPKM GM**	**FPKM WM**	**Fold Change**	**q-value**	**Ensembl ID**
CBLN4	cerebellin 4 precursor			chr20	9.98418	0.0288865	345.6348121	0.00290815	ENSG00000054803
-	intergenic, 1 exon, 5391bps			chr10	1.04349	0.00475891	219.2707994	0.0473629	not annotated
KCNS2	potassium voltage-gated channel, delayed-rectifier, subfamily S, member 2			chr8	1.56421	0.00718485	217.7094859	4.20E-06	ENSG00000156486
SDR16C5	short chain dehydrogenase/reductase family 16C, member 5			chr8	1.46311	0.00753222	194.2468489	0.0200461	ENSG00000170786
KIAA1239	KIAA1239			chr4	1.12395	0.00660563	170.1503112	0.000270041	ENSG00000174145
GLRA3	glycine receptor, alpha 3			chr4	1.31853	0.00902946	146.0253437	0.000379934	ENSG00000145451
C1QL3	complement component 1, q subcomponent-like 3			chr10	6.69439	0.0476218	140.5740648	0.00597669	ENSG00000165985
-	intergenic, 2 splice variants: 1st: 1 exon, 3048 bps 2nd: 2 exons, 2469 bps			chr17	2.88395	0.0245143	117.6435795	0.00298648	not annotated
FLJ41278	uncharacterized LOC400046			chr12	1.74655	0.0150581	115.9874088	0.000318564	ENSG00000255693
SLC17A6	solute carrier family 17 (sodium-dependent inorganic phosphate cotransporter), member 6			chr11	2.44851	0.0226146	108.2712053	0.00269117	ENSG00000091664
**Up-regulated in WM**									
**Gene**	**Description**			**Chrom.**	**FPKM GM**	**FPKM WM**	**Fold Change**	**q-value**	**Ensembl ID**
SLC47A2	solute carrier family 47, member 2			chr17	0.0592195	4.7187	79.68152382	0.00109939	ENSG00000180638
-	intergenic, 1 exon, 6039 bps			chr7	0.0201173	1.58368	78.72229375	0.04031	not annotated
CCDC19	coiled-coil domain containing 19			chr1	0.0212159	1.35457	63.84692613	0.0248006	ENSG00000213085
LINC00162	long intergenic non-protein coding RNA 162			chr21	0.0968483	5.6667	58.51109415	0.00926851	ENSG00000224930
SERPINA5	serpin peptidase inhibitor, clade A (alpha-1 antiproteinase, antitrypsin), member 5			chr14	0.0486903	1.87777	38.56558699	0.00467642	ENSG00000188488
-	intergenic, 2 splice varaints, both 2 exons. 1st: 3294 bps 2nd: 356 bps			chr5	0.0833172	2.88574	34.63558545	0.0163335	not annotated
DAO	D-amino-acid oxidase			chr12	0.180005	6.11662	33.98027833	0.000196888	ENSG00000110887
-	intergenic, 2 exons, 6042 bps			chr17	0.0364359	1.18023	32.39195409	0.0345805	not annotated
FFAR3	free fatty acid receptor 3			chr19	0.0459175	1.13566	24.73261828	0.0496471	ENSG00000185897
LTF	lactotransferrin			chr3	0.520221	12.5738	24.17011232	4.14E-06	ENSG00000012223

Comparing the entirety of the lists, there were more lincRNAs in WM than GM (3 to 1). While there were more unannotated up-regulated genes in GM (80) than WM (57), the unannotated genes in WM represented a greater proportion of the up-regulated genes in WM than the proportion of up-regulated genes in GM represented by unannotated genes. A chi-squared statistical test was performed to determine if there was any statistically significant difference between the proportions of unannotated genes in each of the data sets. The chi-squared test conclusively showed that the difference in the proportions of unannotated genes in each data set was greater than what would be expected by chance alone (p<0.00005), suggesting that, overall, there was a statistically significant higher proportion of unannotated genes identified in WM.

**Figure 2:**
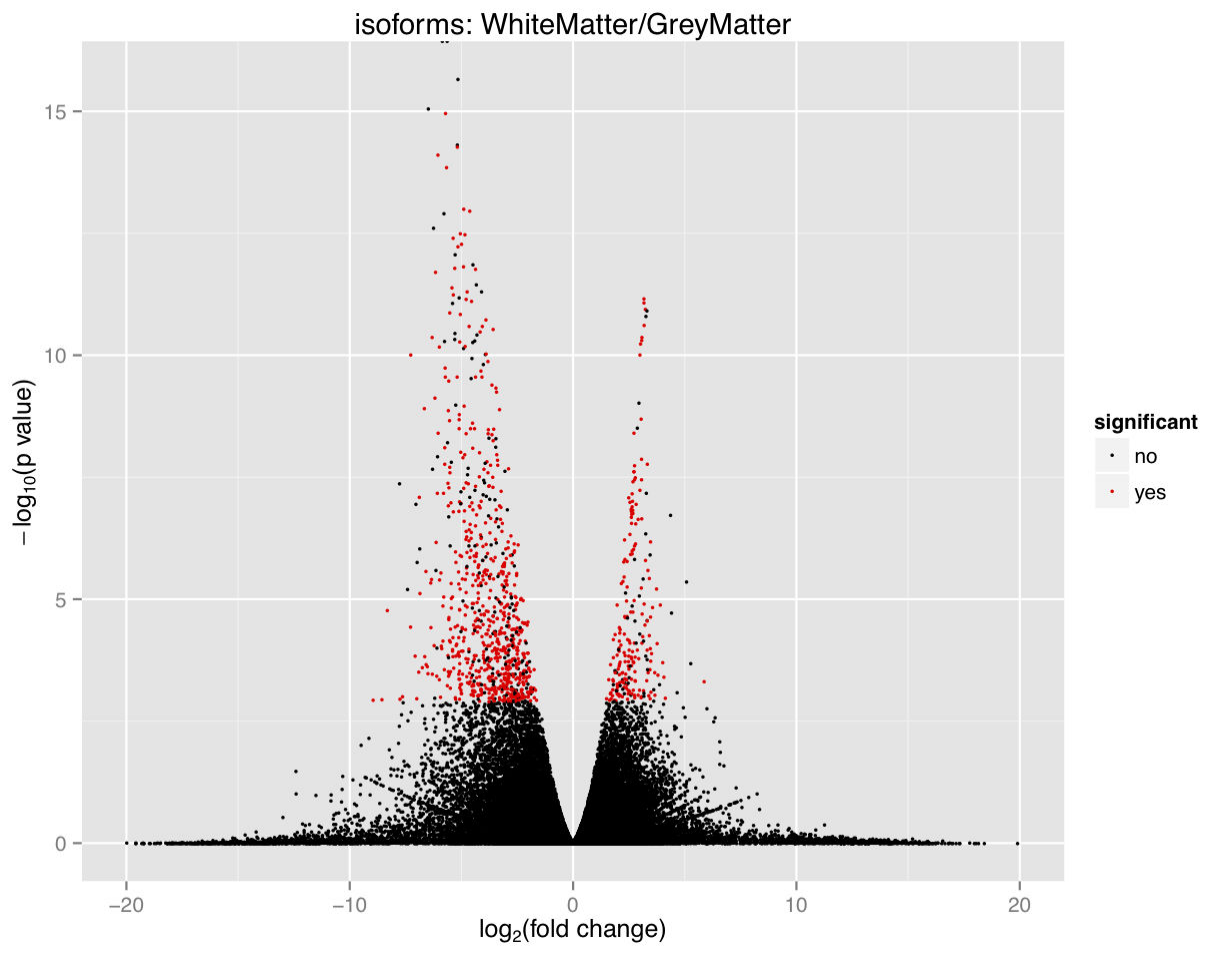
Volcano plot of isoform expression in GM and WM. The fold-change of the isoforms was relative to their expression in WM. Those isoforms with a negative fold-change were up-regulated in GM (down-regulated in WM) and those isoforms with a positive fold-change were up-regulated in WM (down-regulated in GM). Isoforms that were statistically significant (q-value<0.05) are shown in red and were listed in Table S2. This figure demonstrates that a larger number of isoforms were significantly up-regulated in GM (681) than were up-regulated in WM (201). Overall there was a greater spread of data for the isoforms that were up-regulated in GM, both in terms of fold-change and p-values.

Cuffdiff identified 36,306 isoforms in both GM and WM, including 3844 unannotated isoforms (Figure 2). Of this set, 882 isoforms were significantly differentially expressed between GM and WM (q-value<0.05), including 681 isoforms up-regulated in GM and 201 up-regulated in WM (Table S2). When the criteria of having at least one condition with an FPKM>1 was applied this list was reduced to a total of 856 isoforms, with 657 isoforms up-regulated in GM and 199 up-regulated in WM. The top ten differentially expressed isoforms sorted by fold-change from GM and WM respectively are listed in Table 2. This list shares almost no commonality with Table 1; only *C1QL3* and *FLJ41278* (up-regulated in GM) appear in both tables. All of the top 10 up-regulated isoforms in WM are unique to WM and have no expression in GM (Table 2). In fact, the top 24 up-regulated isoforms in WM were not detected at all in GM; in contrast, there was only one unique up-regulated isoform present in GM (Table S2). 

**Table 2:** Top 10 up-regulated isoforms in GM and WM.

**Up-regulated in GM**									
**Gene**	**Description**			**Chrom.**	**FPKM GM**	**FPKM WM**	**Fold Change**	**q-value**	**Ensembl ID**
KCNK12	potassium channel, subfamily K, member 12			chr2	2.81439	0	Unique to GM	0.0441582	ENSG00000184261
OLFM3	olfactomedin 3			chr1	2.6659	0.00538715	494.8627753	0.0477668	ENSG00000118733
KCNC1	potassium voltage-gated channel, Shaw-related subfamily, member 1			ch 11	12.8201	0.0341285	375.6420587	0.0473807	ENSG00000129159
RIMS3	regulating synaptic membrane exocytosis 3			chr1	28.2837	0.14296	197.8434527	0.0423304	ENSG00000117016
C1QL3	complement component 1, q subcomponent-like 3			chr10	6.69439	0.0433715	154.3499764	0.00357408	ENSG00000165985
GABRB2	gamma-aminobutyric acid (GABA) A receptor, beta 2			chr5	17.313	0.112452	153.9590225	8.69E-08	ENSG00000145864
RAB27B	RAB27B, member RAS oncogene family			chr18	3.83441	0.0286122	134.0131133	0.0100352	ENSG00000041353
GALNT9	UDP-N-acetyl-alpha-D-galactosamine:polypeptide N-acetylgalactosaminyltransferase 9 (GalNAc-T9)			chr12	15.68	0.123142	127.3326728	0.0454933	ENSG00000182870
CAMK2A	calcium/calmodulin-dependent protein kinase II alpha			chr5	79.8284	0.664104	120.2046667	0.018091	ENSG00000070808
RGS4	regulator of G-protein signaling 4			chr1	56.2238	0.476735	117.9351212	2.53E-05	ENSG00000117152
**Up-regulated in WM**									
**Gene**	**Description**			**Chrom.**	**FPKM GM**	**FPKM WM**	**Fold Change**	**q-value**	**Ensembl ID**
EIF3F	eukaryotic translation initiation factor 3, subunit F			chr11	0	16.3903	Unique to WM	0.00528201	ENSG00000175390
SUPT5H	suppressor of Ty 5 homolog (*S. cerevisiae*)			chr19	0	13.427	Unique to WM	0.0474906	ENSG00000196235
KIFAP3	kinesin-associated protein 3			chr1	0	8.74404	Unique to WM	0.000345075	ENSG00000075945
ATP6V1H	ATPase, H+ transporting, lysosomal 50/57kDa, V1 subunit H			chr8	0	8.14233	Unique to WM	0.00382367	ENSG00000047249
FLNB	filamin B, beta			chr3	0	7.70063	Unique to WM	0.00648399	ENSG00000136068
PAK2	p21 protein (Cdc42/Rac)-activated kinase 2			chr3	0	6.18142	Unique to WM	0.00233423	ENSG00000180370
ZDHHC3	zinc finger, DHHC-type containing 3			chr3	0	6.10182	Unique to WM	0.0064209	ENSG00000163812
SEZ6L2	seizure related 6 homolog (mouse)-like 2			chr16	0	5.73869	Unique to WM	0.0315557	ENSG00000174938
DCAF7	DDB1 and CUL4 associated factor 7			chr17	0	4.83543	Unique to WM	0.0155276	ENSG00000136485
MECP2	methyl CpG binding protein 2 (Rett syndrome)			chrX	0	4.18897	Unique to WM	0.00357408	ENSG00000169057

### G protein-coupled receptor 123

The G protein-coupled receptor 123 (*GPR123*) gene had one such isoform that was uniquely expressed in WM. However, when analyzed as an entire gene, *GPR123* was found to be 5-fold up-regulated in GM, with overall expression levels of 10 FPKM in GM and 2 FPKM in WM. Further investigation revealed unique splicing patterns that underline the importance of analyzing genes at the isoform level. 

**Figure 3:**
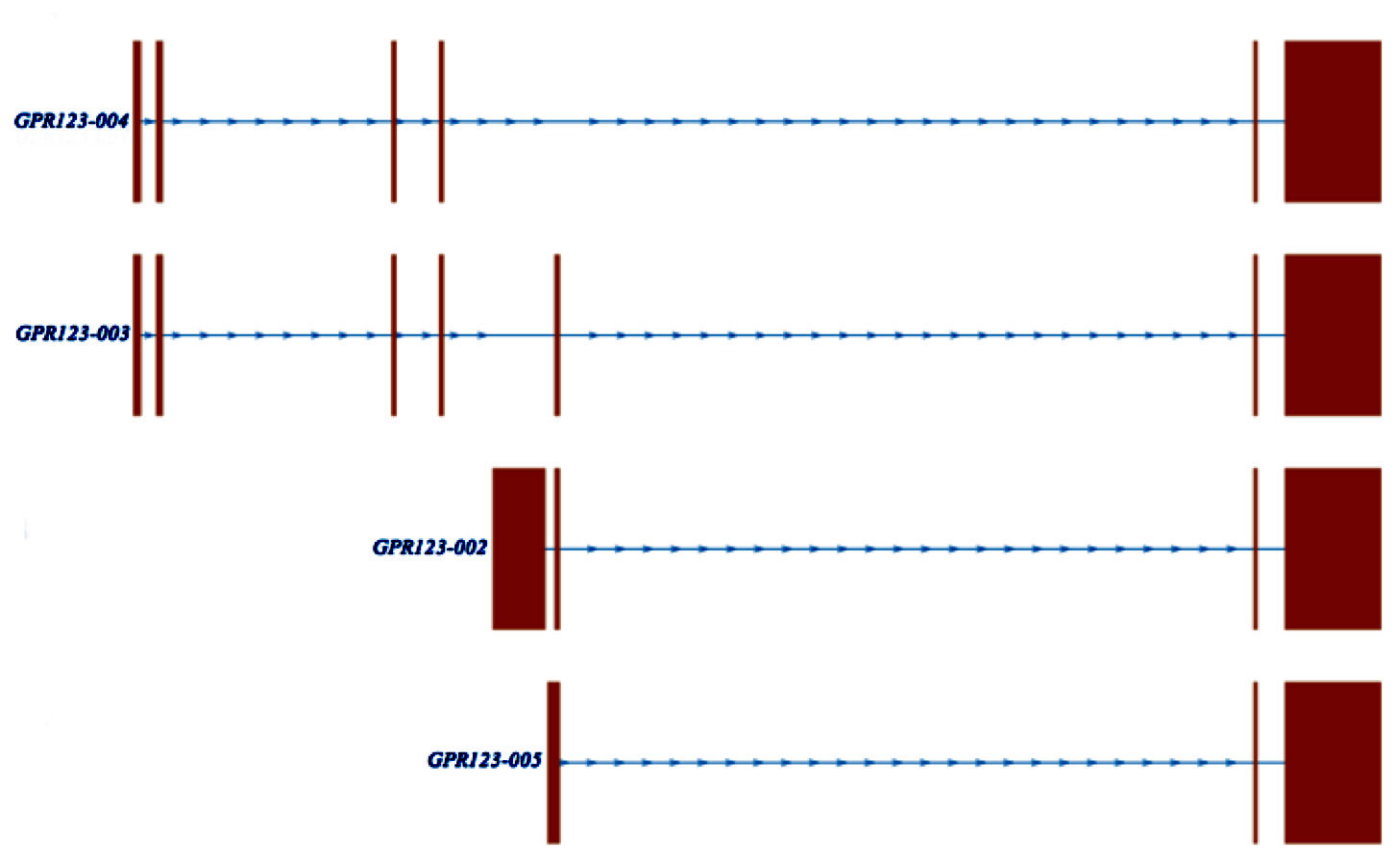
Splice variants of *GPR123*. The intron/exon structure of the four *GPR123* isoforms. *GPR123-002* and *GPR123-003* have been identified previously. *GPR123-003* is considered the full-length isoform. *GPR123-004* and *GPR123-005* were previously unannotated.

Overall, there were four splice variants identified in both GM and WM, including two previously identified splice variants—*GPR123-002* (ENST00000392607) and *GPR123-003* (ENST0000039606)—and two novel splice variants—*GPR123-004* and *GPR123-005* (Figure 3). The two previously identified isoforms (*GPR123-002* and *GPR123-003*) are both protein coding; GPR123-003, with its extended N-terminal, can be considered a full-length, fully functional protein. By comparison, *GPR123-002* was missing the first four exons. Its first exon is an untranslated alternate exon. The translation of *GPR123-002* results in a 97-amino acid N-terminal truncated protein. The exon structure of the novel isoform *GPR123-004* was most similar to *GPR123-003*, but it lacks the 5th exon. The lack of this exon is predicated to result in a 199-amino acid truncation at the C-Terminal end of the GPR123-004 protein when compared to GPR123-003. *GPR123-005* was most similar to *GPR123-002*, albeit with a slight modification to the 5’ region. Translation of *GPR123-005* was predicted to result in a protein similar to *GPR123-002*. Among the four *GPR123* isoforms, three different transcription start sites (TSS) were identified and *GPR123-003* and *GPR123-004* had the same TSS, while the two N-terminal truncated isoforms (*GPR123-002* and *GPR123-005*) both utilized unique TSS.

**Figure 4:**
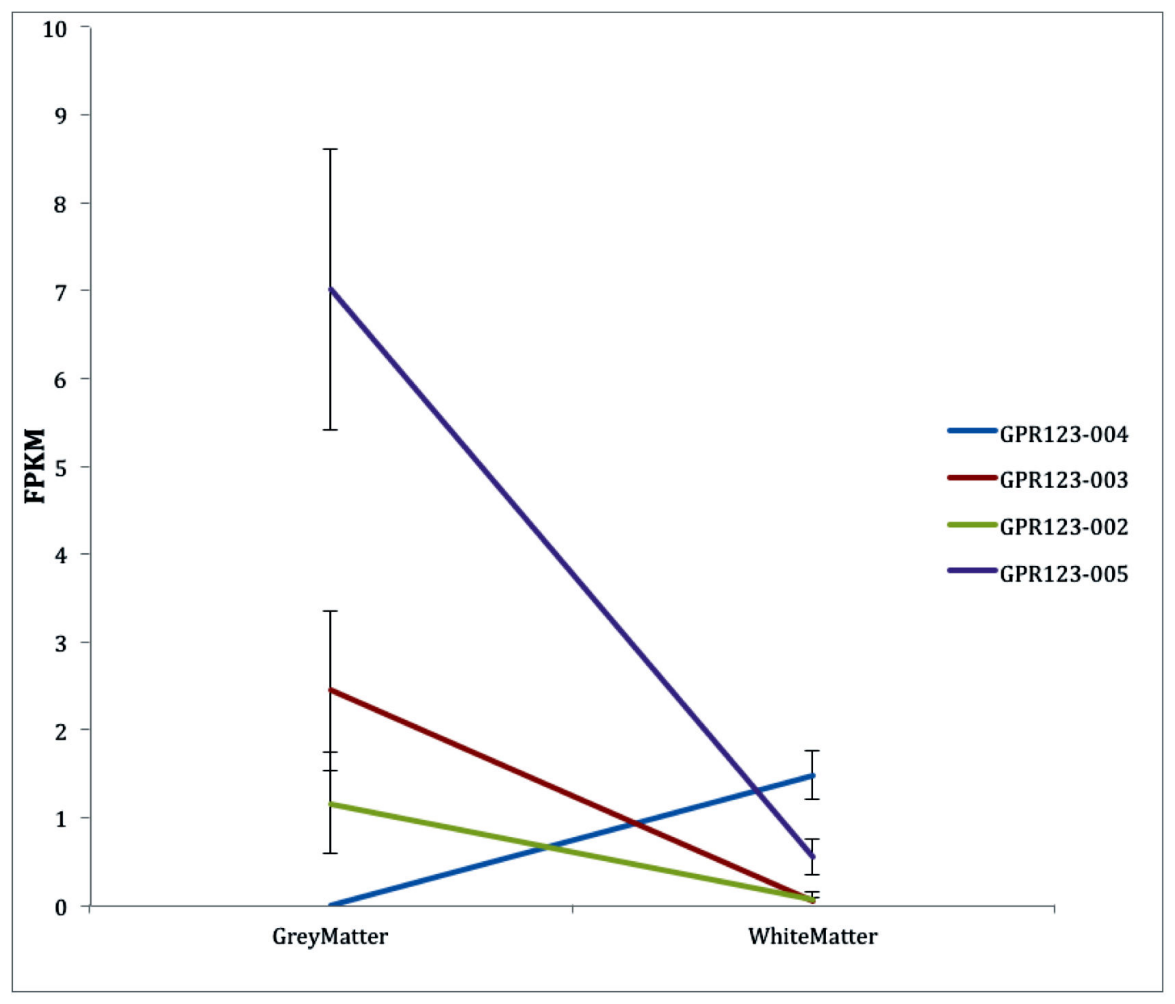
Expression levels of the *GPR123* isoforms. *GPR123-004* was not expressed at all in GM, but became the dominant isoform in WM. *GPR123-002, GPR123-003* and *GPR123-005* all had reduced expressed in WM. The TSS for *GPR123-005* was also up-regulated in GM. The changes in expression of *GPR123-003* and *GPR123-003* were statisitically significant (q-value<0.05). Error bars are ± standard error.

In GM, the dominant isoform was found to be the novel truncated isoform *GPR123-005*. This isoform contributed to approximately 70% of the total *GPR123* expression in GM, and the full length protein coding isoform *GPR123-003* and the truncated protein coding isoform *GPR123-002* contributed to approximately 20% and 10% of the total *GPR123* expression seen in GM, respectively (Figure 4). These three isoforms were all expressed at a higher level in GM when compared to WM, with the 13-fold up-regulation of the *GPR123-005* considered to be statistically significant. The TSS of *GPR123-005* was also up-regulated in GM. *GPR123-004* was not expressed at all in GM. In WM, the three GM expressed isoforms were expressed at very low levels (<0.6 FPKM). In *GPR123-004*, the isoform that was not expressed in GM becomes the dominant isoform, contributing to 70% of all expression of *GPR123* in WM. In this case, the full length protein coding isoform *GPR123-003* contributed to only 1.5% of the total *GPR123* expression. Between GM and WM, there was a switch in the dominant *GPR123* isoforms from *GPR123-005* to *GPR123-004*. Interestingly, although total *GPR123* was expressed at a lower level in WM than GM, the dominant isoform contributed to the same percentage of the overall expression.

The isoform expression patterns of the genes that had the top three annotated differently expressed isoforms in GM and the top three annotated differently expressed isoforms in WM were also analyzed (Figure S2-S7) and shown that the total expression levels of many genes result from dominant expression of one of the isoforms whereas the remaining splice variants are marginally present. Thus it is important to examine gene expression with resolution to individual transcriptional isoforms.

### Expression of well-known cell type markers

While few studies explore the transcriptome profiles of GM and WM, there are a number of well-characterized cell type markers [25]. As the cellular make up of GM and WM is considerably different, certain cell type markers were used to reflect the composition of the respective regions; in this case, established neuronal markers were used as surrogate markers for GM and established oligodendrocyte markers were used as surrogate markers for WM (Table 3). While oligodendrocytes are not entirely specific to WM, they would be expected to appear at a higher expression level in WM than GM.

**Table 3:** Cell type markers.

**Neuronal markers**								
**Gene**	**Description**	**Chrom.**	**FPKM GM**	**FPKM WM**	**Fold Change**	**q-value**	**Significant**	**Ensembl ID**
NEFL	neurofilament, light polypeptide	chr8	204.57	2.89839	70.58056369	1.00E-06	yes	ENSG00000104725
GABRA1	gamma-aminobutyric acid (GABA) A receptor, alpha 1	chr5	18.8275	0.358014	52.58872558	0.00155552	yes	ENSG00000022355
SYT1	synaptotagmin I	chr12	87.9017	1.49415	58.83057257	0.000873923	yes	ENSG00000067715
SLC12A5	solute carrier family 12 (potassium/chloride transporter), member 5	chr20	31.4802	0.808493	38.93688628	1.17E-08	yes	ENSG00000124140
SV2B	synaptic vesicle glycoprotein 2B	chr15	16.0663	1.40407	11.44266312	0.000361367	yes	ENSG00000185518
SNAP25	synaptosomal-associated protein, 25kDa	chr20	974.198	34.271	28.42630796	0.173004	no	ENSG00000132639
KCNQ2	potassium voltage-gated channel, KQT-like subfamily, member 2	chr20	1.31699	0	Unique to GM	0.780598	no	ENSG00000075043
**Oligodendrocyte markers**								
**Gene**	**Description**	**Chrom.**	**FPKM GM**	**FPKM WM**	**Fold Change**	**q-value**	**Significant**	**Ensembl ID**
SOX10	SRY (sex determining region Y)-box 10	chr22	20.4556	133.049	6.504282446	0.0450965	yes	ENSG00000100146
GJC2	gap junction protein, gamma 2, 47kDa	chr1	7.88551	57.9386	7.347476574	4.56E-07	yes	ENSG00000198835
MOG	myelin oligodendrocyte glycoprotein	chr6	8.33271	49.0835	5.890460606	0.000313549	yes	ENSG00000204655
MAG	myelin associated glycoprotein	chr19	62.8597	530.705	8.442690627	1.60E-05	yes	ENSG00000105695
MAL	mal, T-cell differentiation protein	chr2	68.0608	546.616	8.03128967	0.0139824	yes	ENSG00000172005
GAL3ST1	galactose-3-O-sulfotransferase 1	chr22	6.15286	41.0321	6.668784923	0.000107928	yes	ENSG00000128242
UGT8	UDP glycosyltransferase 8	chr4	23.6732	88.8345	3.752534512	0.00966086	yes	ENSG00000174607
CSPG4	chondroitin sulfate proteoglycan 4	chr15	2.95805	4.12994	1.396169774	0.732599	no	ENSG00000173546
PDGFRA	platelet-derived growth factor receptor, alpha polypeptide	chr4	4.67575	4.8763	1.042891515	0.992145	no	ENSG00000134853
MOBP	myelin-associated oligodendrocyte basic protein	chr3	160.105	851.149	5.316192499	0.374118	no	ENSG00000168314

**Figure 5:**
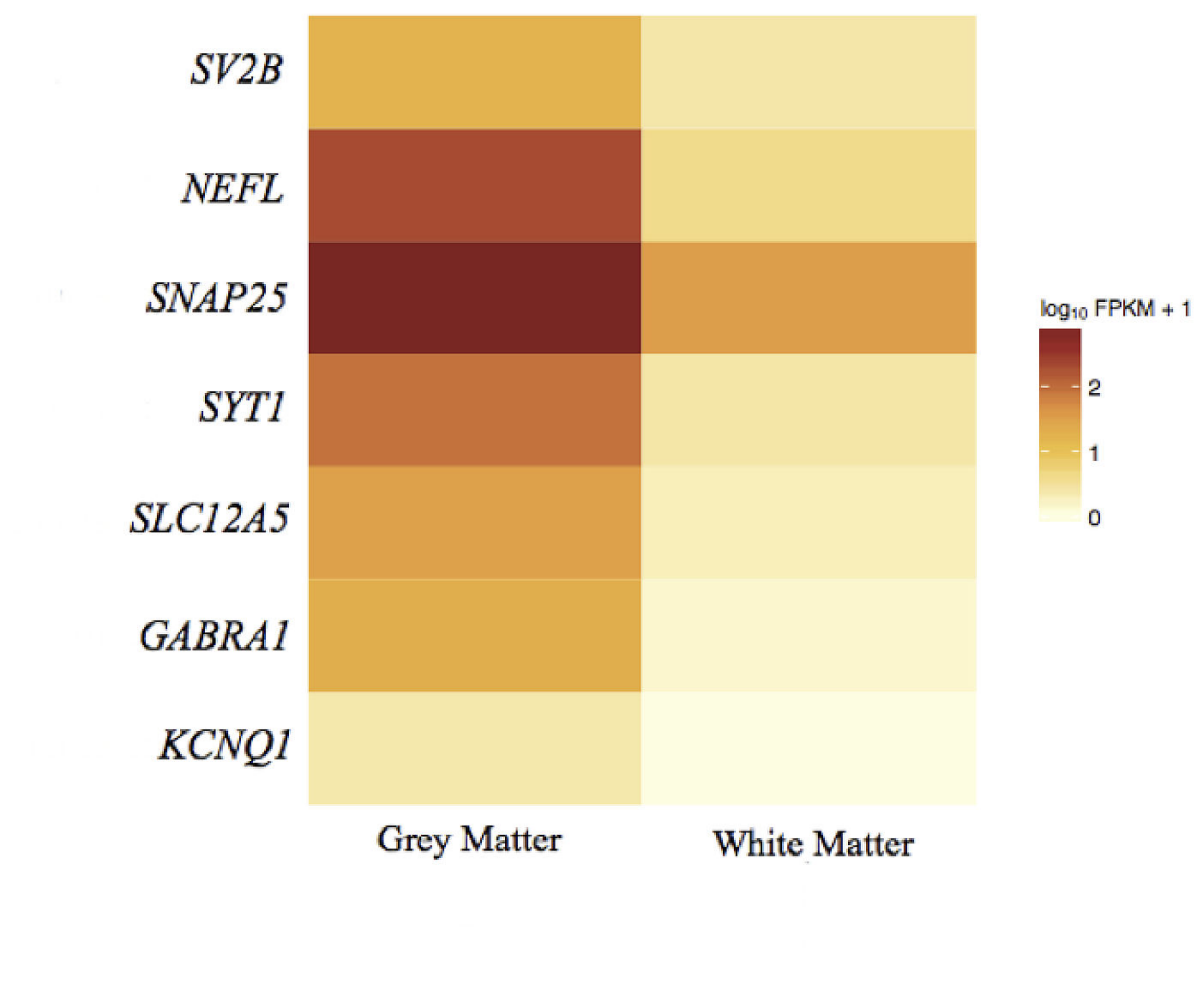
Heatmap of gene expression of neuronal cell markers. Shows the expression profile of neuronal cell markers in GM and WM. There was an expression bias towards GM with the up-regulation of *NEFL, GABRA1, SYT1, SLC12A5, SV2B* being statistically significant (q-value<0.05).

**Figure 6:**
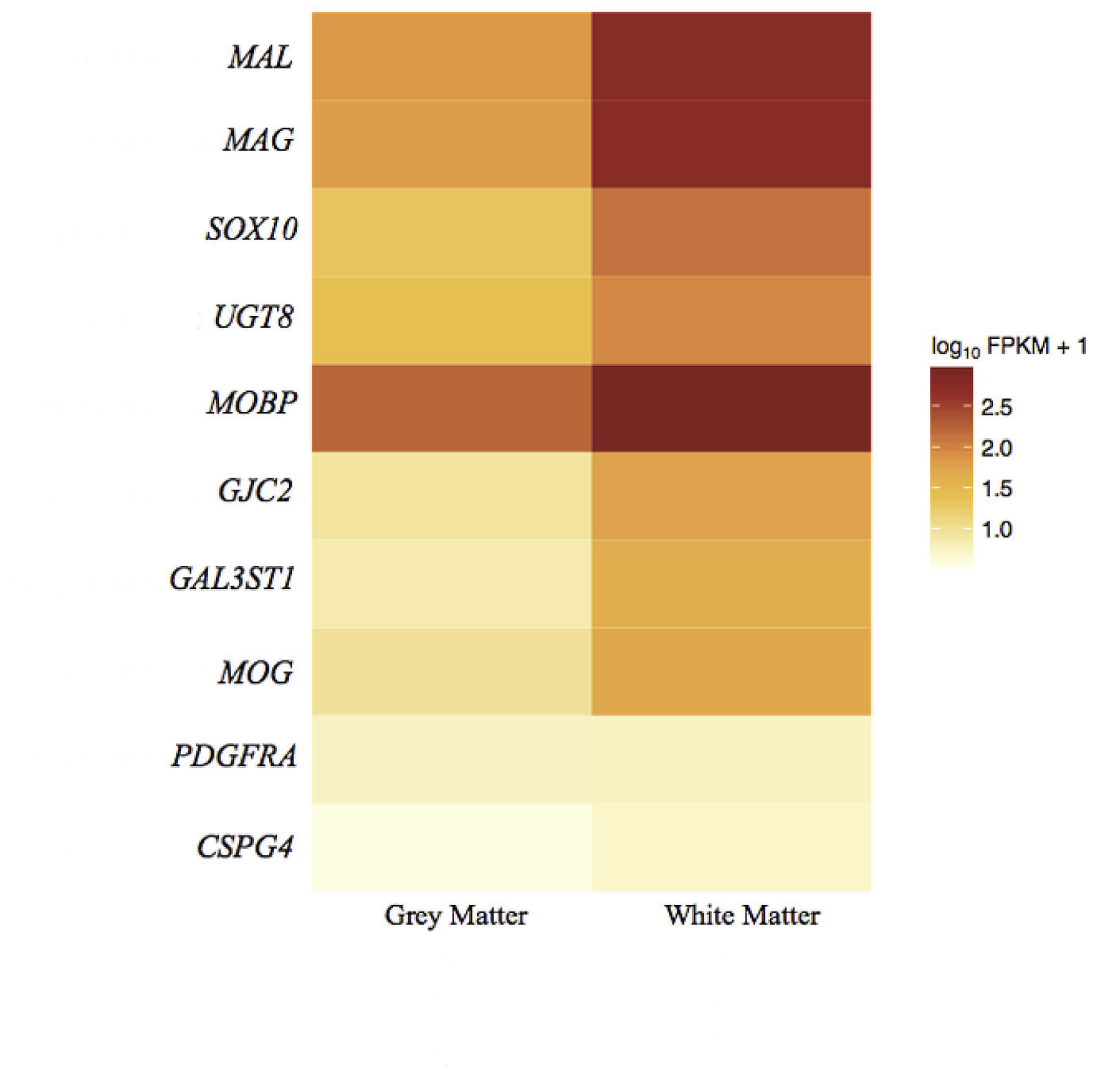
Heatmap of gene expression of oligodendrocyte cell markers. Shows the expression profile of oligodendrocyte cell markers in GM and WM. There was an expression bias towards WM with the up-regulation of *SOX10, GJC2, MOG, MAG, MAL, GAL3ST1, UGT8* being statistically significant (q-value<0.05).

Seven neuronal markers were chosen, all of which were expressed at higher levels in GM than WM. For five of these genes (*NEFL, GABRA1, SYT1, SLC12A5, SV2B*), the up-regulation in GM was statistically significant (q-value<0.05) (Figure 5). The neuronal markers clearly correlate with GM, showing limited to no expression in WM. Ten different oligodendrocyte markers were chosen; all were expressed at a higher level in WM than in GM. Seven of these genes (*SOX10*, *GJC2*, *MOG*, *MAG*, *MAL*, *GAL3ST1*, and *UGT8*) were considered differentially expressed, being up-regulated in WM (q-value<0.05) (Figure 6). These results demonstrate that a correlation exists between the cellular composition of GM and WM and the results produced by the transcriptome sequencing. 

### Validation with in situ hybridization

**Figure 7:**
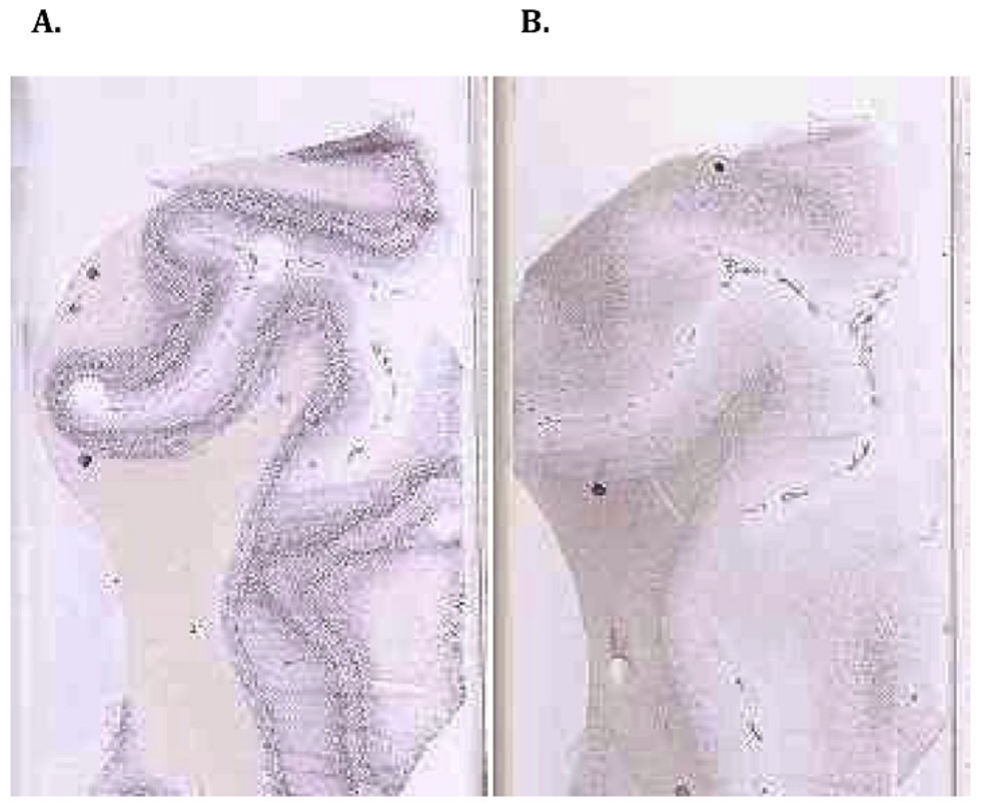
Allen Human Brain Atlas *in* *situ* hybridisation for *NEFH* and *MOG* genes. **A**. *NEFH*: Neurofilament, Heavy Polypeptide GM FPKM: 57.76 WM FPKM: 0.95. Slide from the dorsolateral cortex of a healthy 20-year-old male. The slide shows high levels of expression in GM **B**. *MOG*: Myelin Oligodendrocyte Glycoprotein GM FPKM: 8.33 WM FPKM: 49.08. Slide from the dorsolateral cortex of a healthy 20-year-old male. The slide shows high level of expression in WM. Source: Allen Human Brain Atlas (Hawrylycz et al. 2012 and ©2012 Allen Institute for Brain Science. Allen Human Brain Atlas [Internet]. Available from: http://human.brain-map.org/).

Six genes from the RNA-Seq dataset were selected for further validation through the use of the Allen Brain Atlas ISH database. The first two selected genes were neurofilament heavy polypeptide (*NEFH*) and myelin oligodendrocyte glycoprotein (MOG). *NEFH* encodes for neurofilament-heavy polypeptides, which are present in the chains that form one of the integral components in neuronal cytoskeleton neurofilaments, making it a relevant gene for the neuron rich GM [26,27]. *NEFH* had a FPKM of 57.76 in GM and a FPKM of 0.95 in WM. *MOG* is myelin specific in the central nervous system, and its expression level parallels the myelination of axons, suggesting that it plays an integral role in WM [28]. *MOG* had a FPKM of 8.33 in GM and 49.08 in WM. The ISH results (Figure 7) correlate strongly with the RNA-Seq data. As expected, the *NEFH* ISH slide shows a higher level of expression in GM, while there is almost no expression in WM. Conversely, the ISH slides of *MOG* confirm that *MOG* is expressed at its highest level in WM. The other four genes (*RGS4*, *CAMK2A, SLC17A7, NEFM*) which were selected for validation with *in situ* hybridization also correlated well with the RNA-Seq data (Figure S8). Again, these results demonstrate the accuracy of the RNA-Seq results.

### Novel gene markers for grey matter and white matter

**Figure 8:**
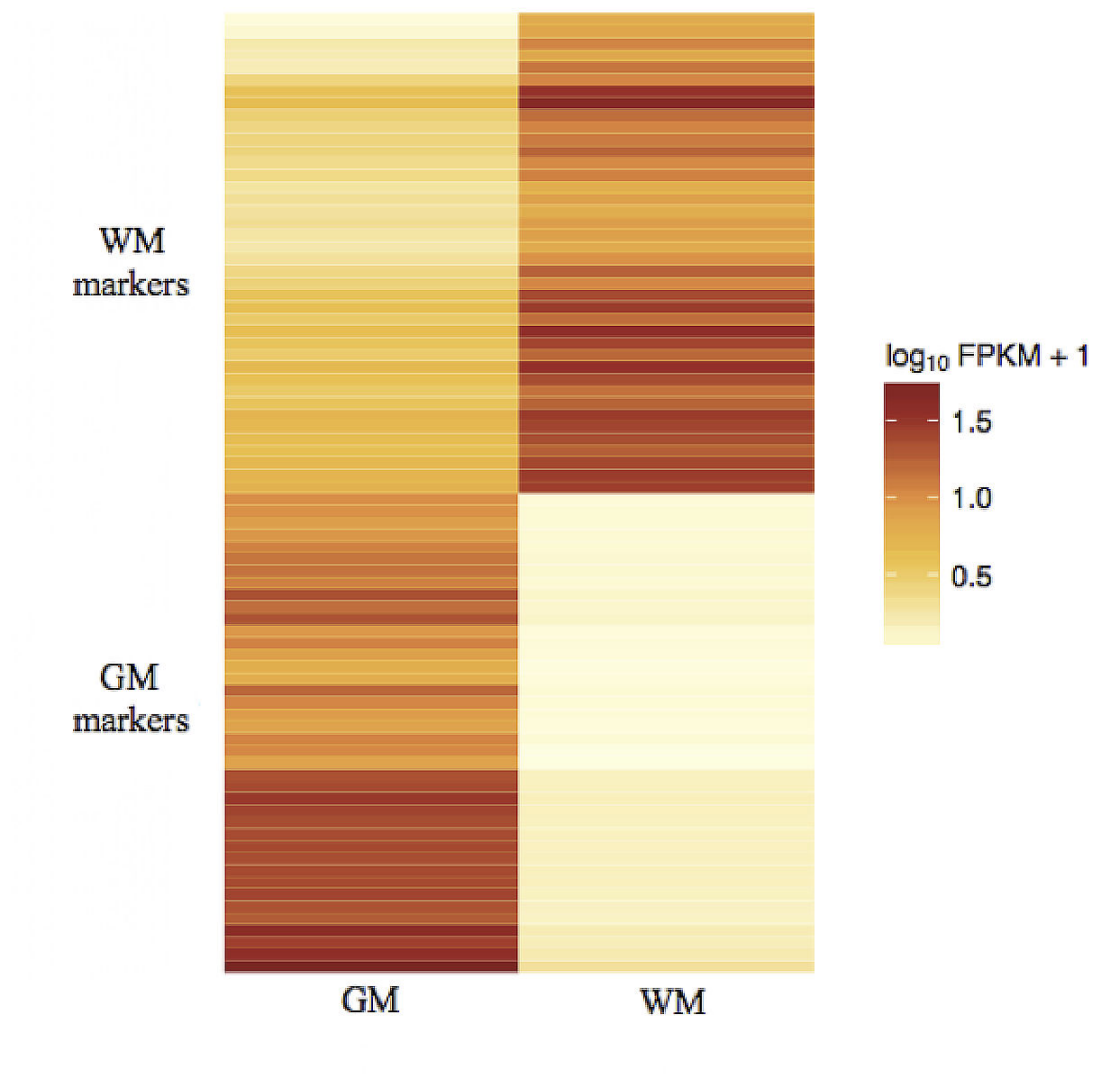
Heatmap of high abundant genes specifically expressed in GM and WM. The following cut-offs were applied to the WM differentially expressed gene list; >4 fold up-regulation in WM, an FPKM>5 in WM and a FPKM <5 in GM. The following cut-offs were applied to the GM differentially expressed gene list; >20-fold up-regulated in GM, had an FPKM>5 in GM and an FPKM<1 in WM. This heatmap shows the top 40 genes from each list. The top half of the heatmap show genes deemed as WM specific, while the lower half shows genes expressed specifically in GM.

Validation of the RNA-Seq results via *in situ* hybridization and correlation with well-known cell type makers demonstrated that RNA-Seq is biologically accurate and thus a useful tool for transcriptome profiling of the human brain. Strict selection criteria were applied to all genes that were up-regulated in GM to define a normal healthy GM transcriptome. The criteria included genes that were >20-fold up-regulated in GM, had an FPKM>5 in GM, and had an FPKM<1 in WM. The aim of this selection stringency was to find genes that were expressed at significantly high levels in GM while not being expressed at all in WM. The criteria reduced the number of genes differentially expressed in GM from 1,218 to 145 (Table S3). As there is less transcriptional activity in WM, the selection criteria were relaxed to the following: >4 fold up-regulation in WM, an FPKM>5 in WM, and a FPKM <5 in GM. These criteria reduced the list of significantly expressed genes in WM from 434 to 76 (Table S3). The top 40 genes from both GM and WM are shown in the form of a heat map (Figure 8). The heat map shows that a marked contrast exists between the refined expression profiles of the two tissue types. In the genes selected as GM markers, the differences between the two tissue types were more distinct than the expression differences seen in the WM. As WM is a less transcriptionally active tissue when compared to GM, WM may experience a flow of RNA from the more transcriptionally active GM. These two gene lists set a baseline for the expression profiles for healthy GM and WM.

### Enrichment map of pathway analysis

The gene lists of differentially expressed genes from GM and WM were fed into DAVID. DAVID sorts genes by gene ontology (GO) terms. The GO terms loosely define the functional relevance of the gene; a gene may belong to numerous ontologies. DAVID then collates the GO terms and determines which ontologies are enriched in the gene list. For GM, a total of 516 different GO terms were considered enriched (Table S4). The top 10 GO terms in GM sorted by p-value are listed in Table 4. For GM, the top 10 GO terms related predominately to synapses and various transport activities. For WM, 284 GO terms were identified as enriched (Table S4). The top 10 GO terms in WM sorted by p-value are listed in Table 4. Of note in this list are the 9th and 10th GO terms, which relate to the ensheathment of neurons and axons.

**Table 4:** Top 10 GO terms in GM and WM.

**GO term GM**	
**Term**	**p-value**
1. Synapse	9.62E-45
2. Synapse part	5.98E-40
3. Synaptic transmission	1.10E-39
4. Transmission of nerve impulse	1.63E-39
5. Plasma membrane part	2.97E-33
6. Gated channel activity	2.38E-30
7. Plasma membrane	3.86E-29
8. Neuron projection	8.53E-28
9. Ion channel complex	1.08E-27
10. Ion channel activity	4.01E-27
**GO terms WM**	
**Term**	**p-value**
1.Plasma membrane	4.78E-06
2. Negative regulation of axonogenesis	7.22E-06
3. Regulation of cell projection organization	9.78E-06
4. Regulation of action potential in neuron	9.83E-06
5. Regulation of cell morphogenesis	1.08E-05
6. Negative regulation of cell projection organization	1.46E-05
7. Regulation of axonogenesis	1.48E-05
8. Ensheathment of neurons	1.61E-05
9. Axon ensheathment	1.61E-05
10. Sterol metabolic process	3.00E-05

**Figure 9:**
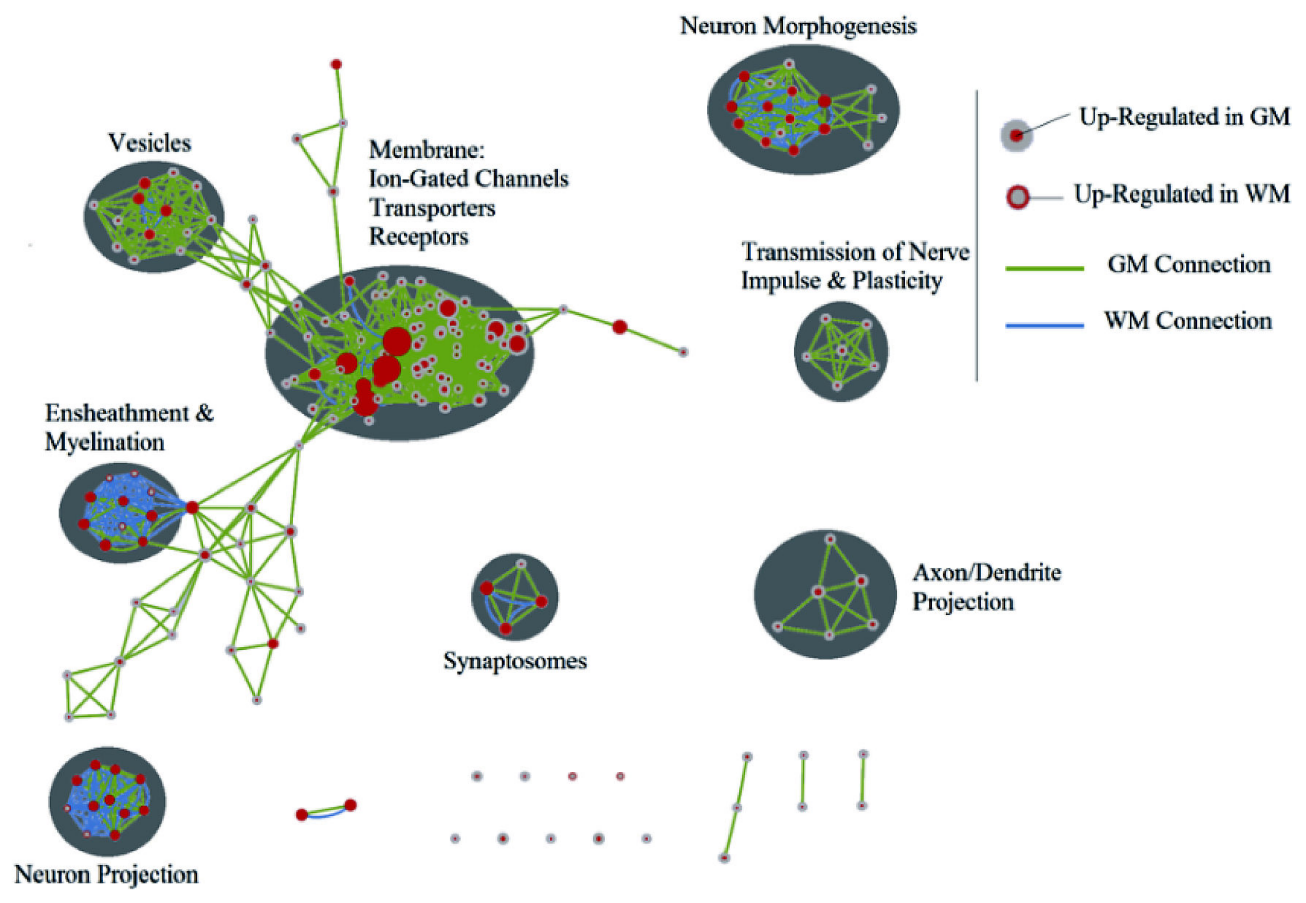
Gene Ontology terms enrichment map for GM and WM transcriptomes. Each node represents a different GO term, the size of the node relates to the level of enrichment of each term. Red in the centre of the node represents up-regulation in GM, red on the edge of the node represents up-regulation in WM. The connections between each GO term are either green or blue. A green connection between node means that the both GO terms are in the GM lists, blue connections represent appearance of the GO terms in the WM lists. The more closely related GO terms are, the closer they appear on the enrichment map. A large number of closely related GO terms forms a cluster. Each cluster has been labelled with a general terms that captures all GO terms.

The list of GO terms from GM and WM were then fed into Cytoscape and used to modify the total gene list, creating an enrichment map (Figure 9). The enrichment map revealed several large and distinct clusters related to vesicles and membrane: ion-gated channels, transporters and receptors, neuron morphogenesis, ensheathment and myelination, neuron projection, synaptosomes, neuron morphogenesis, transmission of nerve impulse, and plasticity and axon/dendrite projection. From the enrichment map, it can be seen that there are more GO terms enriched in GM. Also, there are more GM connections between GO terms, which reinforce the observed trend of higher levels of transcriptional activity in GM than in WM. The major clusters of GO terms for GM related to vesicles and membranes are ion-gated channels, transporters and receptors. These groups are related to the transport of substances such as ions throughout cells. Clusters of GO terms related to neuron morphogenesis and neuron projection were enriched by genes from WM and GM. Neuron morphogenesis refers to changes in the underlying neuronal cytoskeleton and its interaction with the plasma membrane [29]. It can involve processes pertaining to axon initiation, growth, guidance and branching; dendritic growth, guidance, and branching; and synapse formation and stability. Similarly, neuron projection relates to any process involved in the initiation of neurite protrusion, and subsequent elongation often involving axons and dendrites [30]. Neuron morphogenesis and neuron projection are related to improving communication between neurons and hence will involve a complex interaction between GM and WM. Only one cluster was clearly dominated by WM and was related to ensheathment and myelination, a process performed by glia enriched in WM.

## Discussion

This study is the first comparative transcriptome analysis of GM and WM from the human brain using RNA-Seq. It has shown that, overall, there are high levels of transcription in the human brain, with the identified transcripts covering approximately 37% of the genome. This number is close to the figure described by the ENCODE consortium [31]; the ENCODE study reported that, among 15 different human cell lines, the mean coverage of the human genome by primary transcripts was 39%. The ENCODE study included non-polyadenylated RNAs, while the current study utilized poly-T oligo-attached magnetic beads for the selection of the RNA fraction for analysis. If the RNA selection for this study also included non-polyadenylated RNAs, it would be expected that the coverage of the genome would be much higher than the 39% suggested by ENCODE. This would suggest that transcription is pervasive in the human brain. Furthermore, this study has also shown that a number of differentially expressed genes and transcripts exist across GM and WM from the same anatomical region of the human brain, namely, the superior frontal gyrus. These results highlight the complexity and variability of the transcriptome and the presence of numerous non-coding elements. Importantly, the transcriptome profiles for each region, and the GO enrichment map correlated well with what is known about the function and composition of WM and GM. This previous information has been predominately established through the use of other techniques, such as histopathology and *in situ* hybridization. 

The human brain is an extremely complex organ. This complexity is not only derived from the sheer number and variety of cells present in the brain, but also through the heterogeneity of the brain, with cellular composition and density having the propensity to vary over short distances. The tissue samples used for the RNA-Seq analysis were taken from two different tissue types (GM and WM) from adjacent regions of the superior frontal gyrus. While the samples were taken from regions of close proximity, their transcriptome profiles were distinct, suggesting that each of the two tissue types have separate functions and further highlights the heterogeneity of the brain tissue across small distances. The heterogeneity of the brain becomes an issue when attempting to gauge differing gene expression levels between case and control samples. If the brain tissue is not selected and matched properly, differences highlighted in the transcriptome profiles may be differences resulting from variation in the composition and function of different brain regions rather than disease-related changes in the transcriptome. While the heterogeneity of brain tissue could make it difficult to study, the problem can be overcome through appropriate experimental design. The brain regions being compared must have the same cellular composition and be of the same functional capabilities. Laser capture of specific cell populations could be used to create homogenous samples for RNA-Seq analysis [32].

Several previous attempts were made to establish specific gene expression patterns as markers for GM and WM [33,34]. These studies, performed using microarrays, provided only partially overlapping results in terms of GM- and WM-specific genes. These discrepancies are due to the different cortical regions analyzed between studies; thus, the contribution of genes specifically expressed in neurons and oligodendrocytes variably contributed to the calculated GM/WM ratio [3]. Another difficulty stems from the microarray technique itself, which is limited in dynamic range to quantify gene expression [35]. In the present study, we provide a range of over 40 new gene marker candidates based on their unequivocal abundance in expression in GM and WM. Further, we propose that cumulative gene expression signatures, as now commonly used in cancer research [36], rather than individual genes should be used as markers for GM and WM. The utility of such marker signatures should however be further evaluated using larger cohort of samples.

Within the last few years, the RNA-Seq technique became widely used in transcriptome research, progressively replacing microarray techniques. Surprisingly, little has been done in the field of brain diseases using this sequencing approach. This can be partially caused by the lack of comprehensive reference data sets that might be used for comparative transcriptome analysis. Wu and colleagues recently reported comparative analysis of the transcriptome profiles derived from the superior temporal gyrus (STG) [37]. This study was however limited to the GM of STG and performed in the context of schizophrenia. Our study provides the first well-defined data sets that can be used to explore transcriptome aberrations affecting the frontal lobe and, in particular, frontal WM as in the case of multiple system atrophy and bipolar disorders [38,39]. By combining differential gene and isoform expression analysis with pathway analysis we were able to demonstrate that biological processes specific for WM and GM, such as myelination and ions transportation, respectively are reflected on the transcriptional level. Thus the transcriptome profiling may be effectively used to investigate cellular physiology, while a more global view of genomic expression may be used to study phenotypic features of complex structures such as human cortex.

This study identified high levels of various non-coding RNA classes, including snoRNAs, miRNAs, and lincRNAs; further, there were also high levels of locRNAs, novel genes, and novel isoforms produced from splicing events. It is possible that these last three classes could contribute further to the number of non-coding RNAs found in the human brain. Interestingly, while higher levels of transcriptional activity were found in GM, WM was identified as having higher levels of lincRNAs and a higher proportion of unannotated isoforms. Non coding RNAs (ncRNAs) were previously suggested to be widely expressed across the brain, where they often have cell specific regulatory functions [40]. These ncRNAs may play a role in regulating myelination patterns of axon in WM, a dynamic process that can be altered by experience and can carry on for decades in the human brain [41]. The functionality of the unannotated RNAs will need to be explored further in the future. It should also be underlined that possible influence of age on the transcriptional patterns presented here cannot be excluded and will require utilization of larger number of samples to enable regression analysis of covariates.

*GPR123* is a member of the human G protein-coupled receptor (GPCR) family. GPCRs play important roles in a variety of sensory systems and help modulate blood pressure, food intake, immune responses, and development. It has been suggested that *GPR123* is expressed specifically in the central nervous system (CNS); it also shows high levels of conservation across the vertebrate lineage [42]. These factors suggest that *GPR123* expression may be a fundamental component of the vertebrate CNS.

The expression patterns of *GPR123* between GM and WM reveal an interesting individual insight into the complexity of the transcriptome. At the gene level, *GPR123* was up-regulated 5-fold in GM, suggesting that *GPR123* is of greater functional relevance in GM; however, when analyzed at the isoform level, it was shown that the full length isoform *GPR123-003* was not the major contributor to the *GPR123* overexpression. Instead, the dominant isoform was the C-terminal truncated *GPR123-004*, which contributed to approximately 70% of total *GPR123* expression in GM. The level of *GPR123-004* FPKM expression in GM and the use of an alternate TSS suggest that it may be relevant to functional GM. In contrast, the dominant isoform in WM was the N-terminal truncated isoform *GPR123-005*, which contributed approximately 70% of the total *GPR123* expression in WM. It is known that the domain structure of *GPR123-004* and *GPR123-005* would differ, which would lead to distinct functions for proteins encoded by each isoform. This fact points to the possibility that the *GPR123* proteins may carry out distinct tissue-specific functions while being transcribed and translated from the same genomic locus.

While more research needs to be directed at elucidating the role of the differing *GPR123* isoforms in the human brain, this gene does demonstrate the complexity of the transcriptome and also shows how the repertoire of transcripts is greatly increased by splicing events. It also illustrates that it is not just total gene expression levels that are important, but which isoforms are contributing to the expression levels. Unfortunately, current pathway analysis bioinformatic tools, such as DAVID, have not yet been set up to delineate between different splice variants. 

## Conclusions

This study identified a large number of differentially expressed genes between GM and WM that matched up well with previous studies and the known biology of the human cerebrum, underlining the accuracy and the advantages of using RNA-Seq for the analysis of complex transcriptomes. This set of results form a baseline expression database for healthy GM and WM and can be used as a resource to help detect abnormal gene expression between GM and WM tissues from sufferers of neurodegenerative diseases or psychiatric disorders. This is particularly relevant to WM which may play a major role in AD and multiple system atrophy [43,44].

## Supporting Information

Table S1 **Full list of significant DEGs between GM and WM.**
(XLSX)

Table S2 **Full list of significant DEIs between GM and WM.**
(XLSX)

Table S3 **Novel gene markers for GM and WM.**
(XLSX)

Table S4 **Gene Ontology terms enrichment analysis for DEGs between GM and WM.**
(XLSX)

Figure S1 **Gene body coverage for GM and WM RNA samples used in this study.** **A** - **C**. GM samples D - F. WM samples. Each figure shows the number of reads that map to particular portions of the gene body. The x-axis starts at the 5’ end of the transcript and moves towards the 3’ end (left to right). The y-axis represents the average wigsum. The wigusm is a normalised ‘total read count’ where a wigsum of 100,000,000 is equal to the coverage achieved by 1 million 100 base reads or 2 million 50 base reads. All figures are skewed to the 3’ end of the transcripts, showing a 3’ bias, caused by poly-A selection of the RNA fraction.
(TIFF)

Figure S2 **Expression levels of visinin-like 1 (*VSNL1*) isoforms.** There were two *VSNL1* splice variants expressed across GM and WM. *VSNL1-001* was up regulated 34x in GM when compared to WM. The second splice variant *VSLN1-007* was novel and was expressed at low levels across both GM and WM. Error bars are ± standard error.
(TIFF)

Figure S3 **Expression levels of syniclein, beta (*SNCB*) isoforms.** There were two *SNCB* splice variants expressed across GM and WM. *SNCB-002* was the dominant isoform and was up regulated 29x in GM when compared to WM. The second splice variant *SNCB-001* was expressed at low levels across both GM and WM. Error bars are ± standard error.
(TIFF)

Figure S4 **Expression levels of reticulon 1 (*RTN1*) isoforms.** There were four *RTN1* splice variants expressed across GM and WM. *RTN1-202* was the dominant isoform and was up regulated 10x in GM when compared to WM. The splice variant *RTN1-201* was expressed at approximately 20 FPKM in both conditions. *RTN1-203* and *RTN1-204* were expressed at low levels in both conditions. All four identified splice variants were novel. Error bars are ± standard error.
(TIFF)

Figure S5 **Expression levels of transferrin (TF) isoforms.** There were two *TF* splice variants expressed across GM and WM. Both splice vairants contributed almost equal levels of expression to both GM and WM. *TF-001* was upregulated 7x in WM when compared to GM. *TF-202* was a novel splice varaints, it was expressed at higher levels in WM than in GM, however the changes in expression was not considered to be statistically significant. Error bars are ± standard error.
(TIFF)

Figure S6 **Expression levels of myelin associated glycoprotein (MAG) isoforms.** There were four *MAG* splice variants expressed across GM and WM. *MAG-001* was the dominant isoform and was up regulated 8x in WM when compared to GM. The three other splice variants (*MAG-002*, *MAG-003*, *MAG-009*) were expressed at low levels in both GM and WM. *MAG-009* was a novel splice variant. Error bars are ± standard error.
(TIFF)

Figure S7 **Expression levels of MARCKS-like 1 (*MARCKSL1*) isoforms.** There were two *MARCKSL1* splice variants expressed across GM and WM. Both splice vairants contributed high levels of expression to both GM and WM. *MARKSL1-001* was upregulated 7x in WM when compared to GM. *MARCKSL1-002* was a novel splice varaints, it was also expressed at higher levels in WM than in GM, however the changes in expression was not considered to be statistically significant. Error bars are ± standard error.
(TIFF)

Figure S8 **Allen Human Brain Atlas *insitu* hybridisation for the *RGS4*, *CAMK2A, SLC17A7* and *NEFM* genes.** **A**. *RGS4*: Regulator of G-protein signalling 4 GM FPKM: 100.75 WM FPKM: 1.52. Slide from the dorsolateral cortex of a healthy 20-year-old male. The slide shows high levels of expression in GM. **B**. *CAMK2A*: Calcium/calmodulin-dependent protein kinase II alpha GM FPKM: 233.16 WM FPKM: 5.27. Slide from the dorsolateral cortex of a healthy 20-year-old male. The slide shows high levels of expression in GM. **C**. *SLC17A7*: Solute carrier family 17 (sodium-dependent inorganic phosphate cotransporter), member 7 GM FPKM: 270.79 WM FPKM: 7.39. Slide from the dorsolateral cortex of a healthy 20-year-old male. The slide shows high levels of expression in GM. **D**. *NEFM*: Neurofilament, medium polypeptide GM FPKM: 224.01 WM FPKM: 4.09. Slide from the dorsolateral cortex of a healthy 20-year-old male. The slide shows high levels of expression in GM. Source: Allen Human Brain Atlas (Hawrylycz et al. 2012 and ©2012 Allen Institute for Brain Science. Allen Human Brain Atlas [Internet]. Available from: http://human.brain-map.org/).
(TIFF)
