# Supplementary figures and images for: Unique Transcriptome Patterns of the White and Grey Matter Corroborate Structural and Functional Heterogeneity in the Human Frontal Lobe
Source: PLoS One. 2013 Oct 23;8(10):e78480. doi: 10.1371/journal.pone.0078480 (PMC3808538; doi:10.1371/journal.pone.0078480)

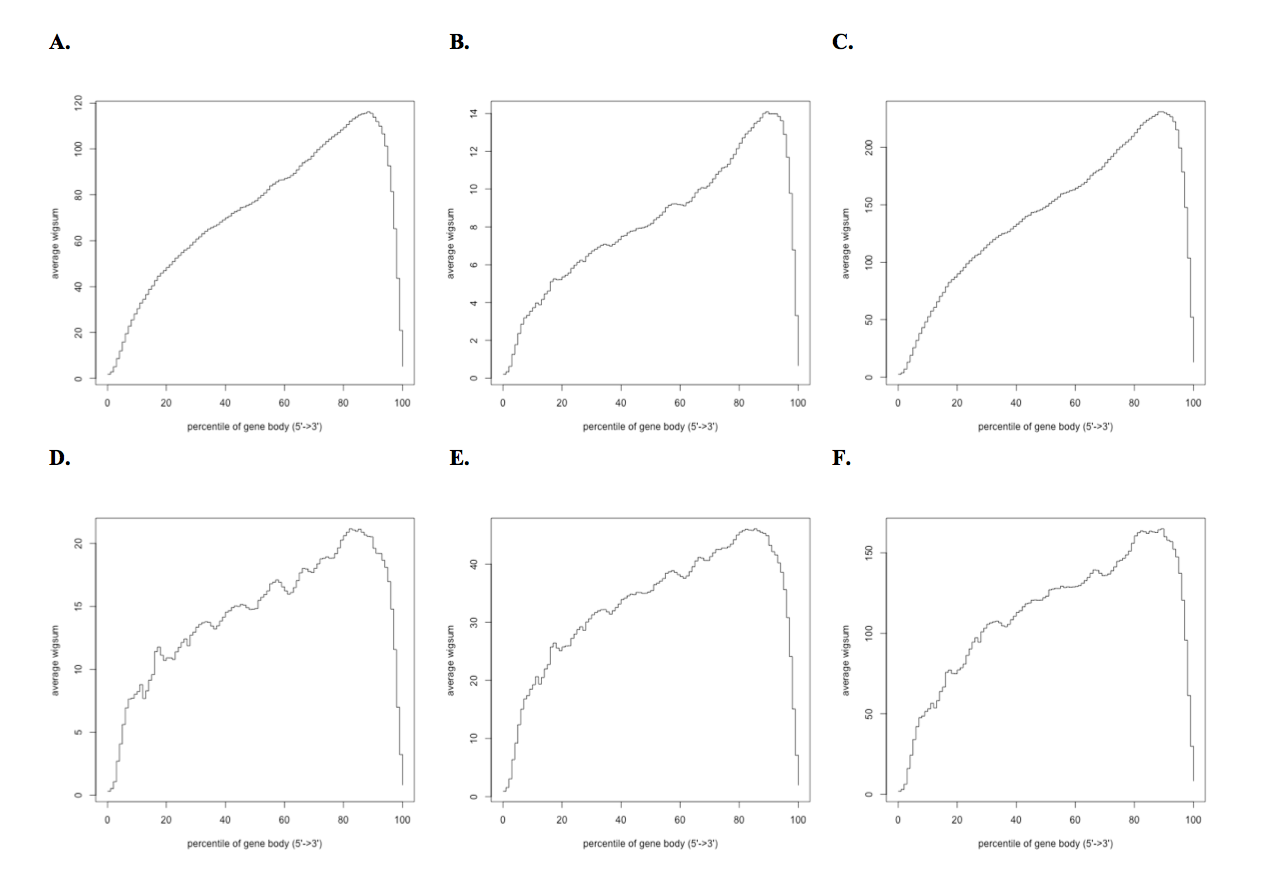

Supplement: Figure S1 — Gene body coverage for GM and WM RNA samples used in this study. A - C. GM samples D - F. WM samples. Each figure shows the number of reads that map to particular portions of the gene body. The x-axis starts at the 5’ end of the transcript and moves towards the 3’ end (left to right). The y-axis represents the average wigsum. The wigusm is a normalised ‘total read count’ where a wigsum of 100,000,000 is equal to the coverage achieved by 1 million 100 base reads or 2 million 50 base reads. All figures are skewed to the 3’ end of the transcripts, showing a 3’ bias, caused by poly-A selection of the RNA fraction. (TIFF) [file pone.0078480.s005.tiff]

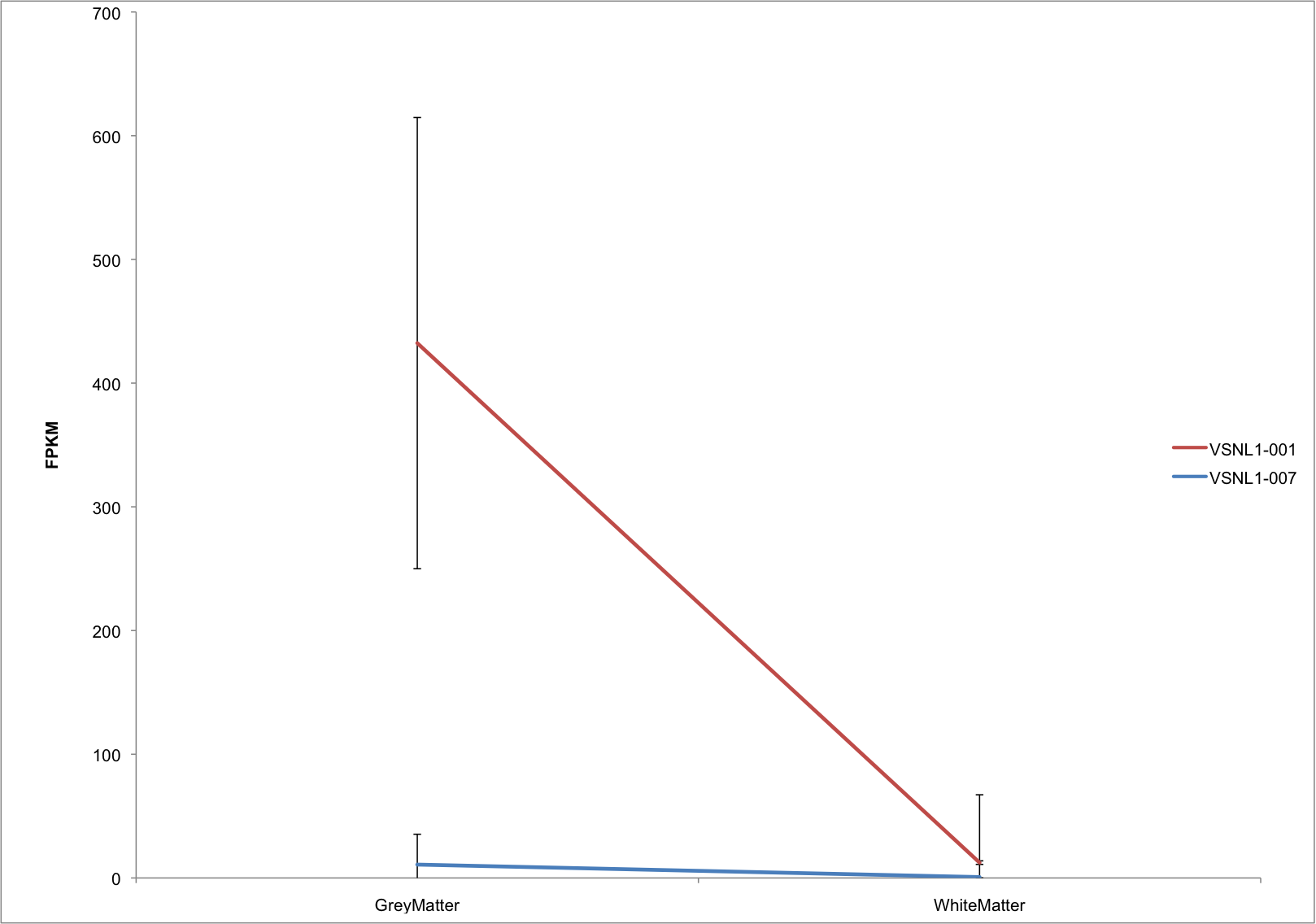

Supplement: Figure S2 — Expression levels of visinin-like 1 (VSNL1) isoforms. There were two VSNL1 splice variants expressed across GM and WM. VSNL1-001 was up regulated 34x in GM when compared to WM. The second splice variant VSLN1-007 was novel and was expressed at low levels across both GM and WM. Error bars are ± standard error. (TIFF) [file pone.0078480.s006.tiff]

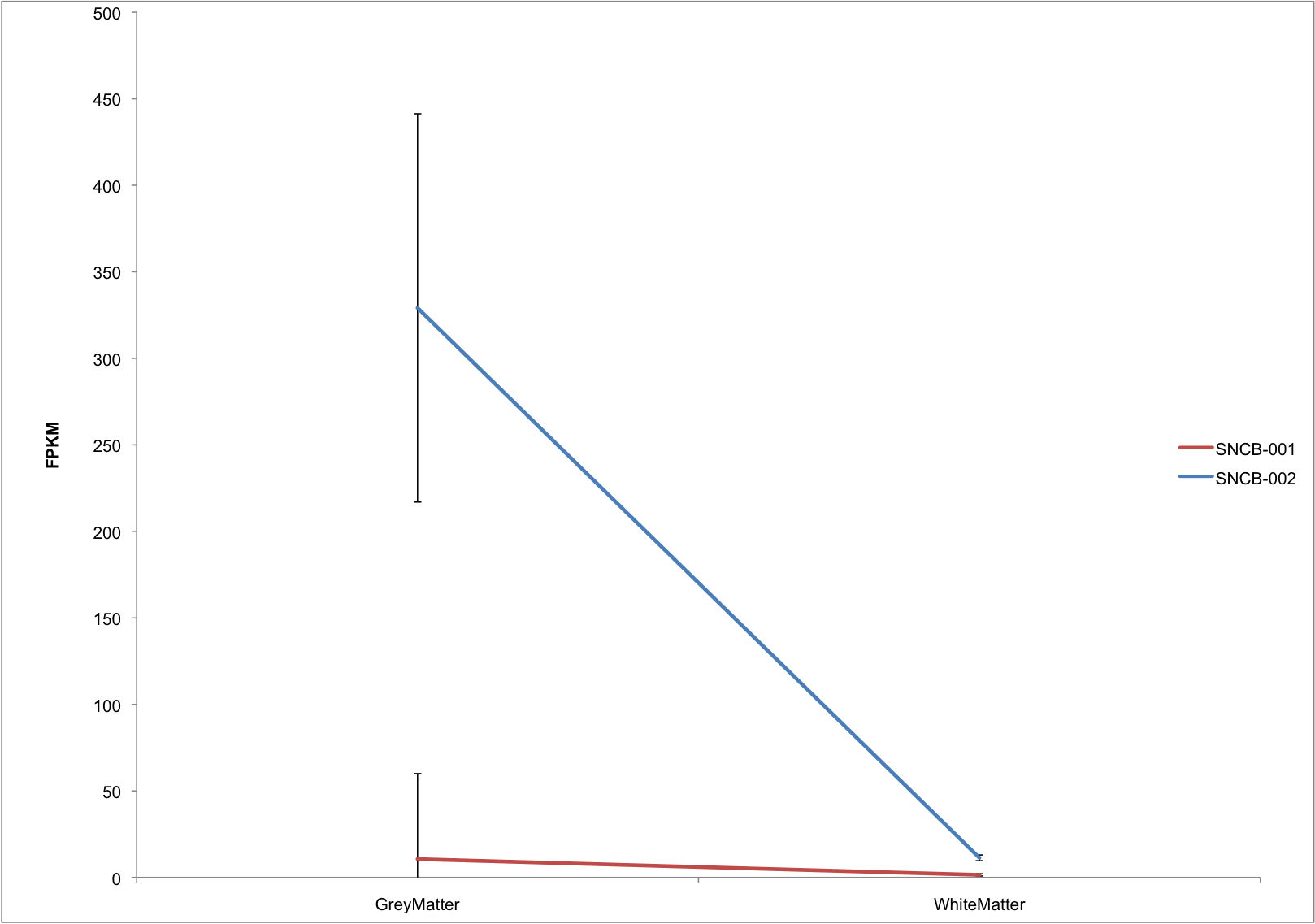

Supplement: Figure S3 — Expression levels of syniclein, beta (SNCB) isoforms. There were two SNCB splice variants expressed across GM and WM. SNCB-002 was the dominant isoform and was up regulated 29x in GM when compared to WM. The second splice variant SNCB-001 was expressed at low levels across both GM and WM. Error bars are ± standard error. (TIFF) [file pone.0078480.s007.tiff]

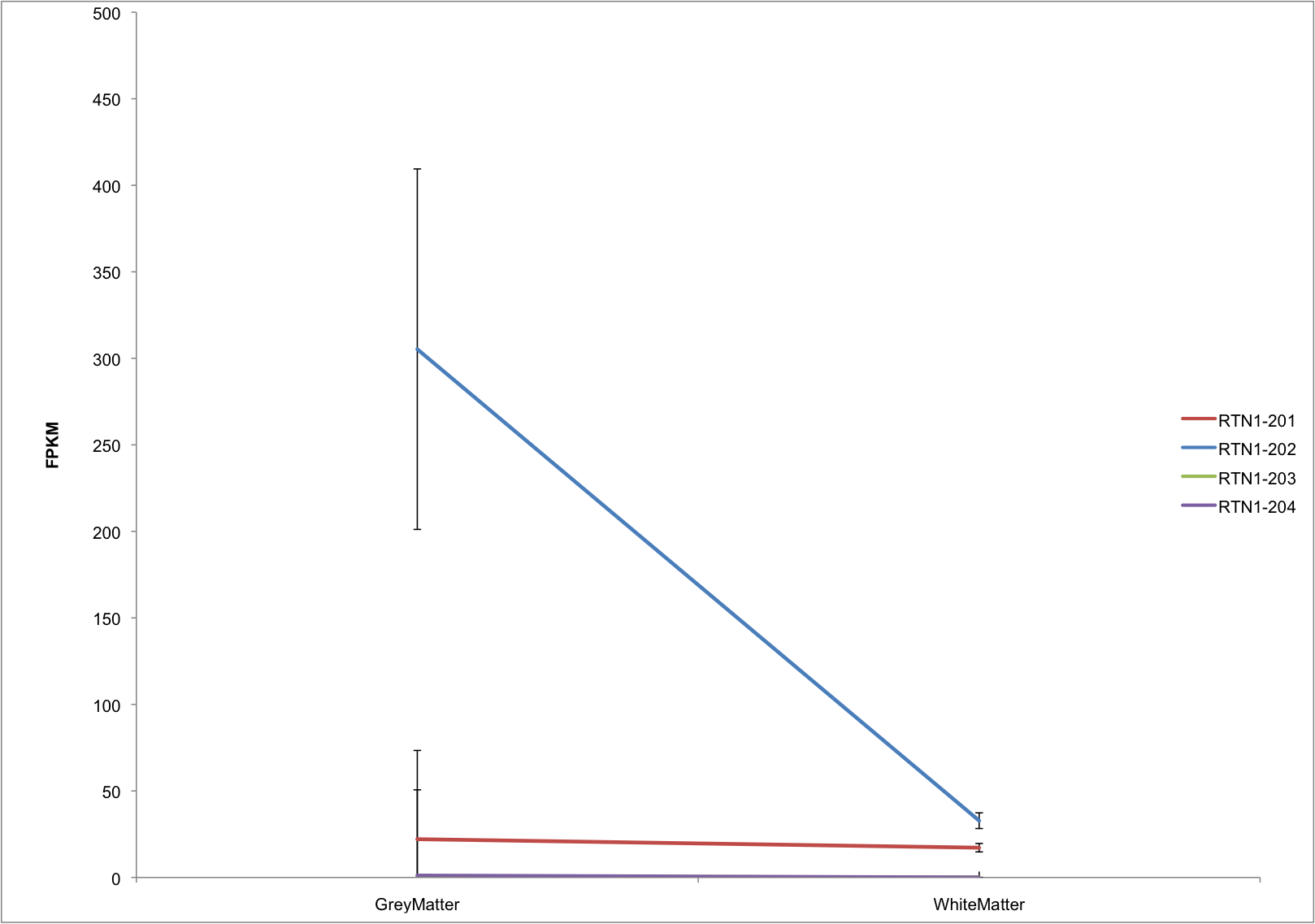

Supplement: Figure S4 — Expression levels of reticulon 1 (RTN1) isoforms. There were four RTN1 splice variants expressed across GM and WM. RTN1-202 was the dominant isoform and was up regulated 10x in GM when compared to WM. The splice variant RTN1-201 was expressed at approximately 20 FPKM in both conditions. RTN1-203 and RTN1-204 were expressed at low levels in both conditions. All four identified splice variants were novel. Error bars are ± standard error. (TIFF) [file pone.0078480.s008.tiff]

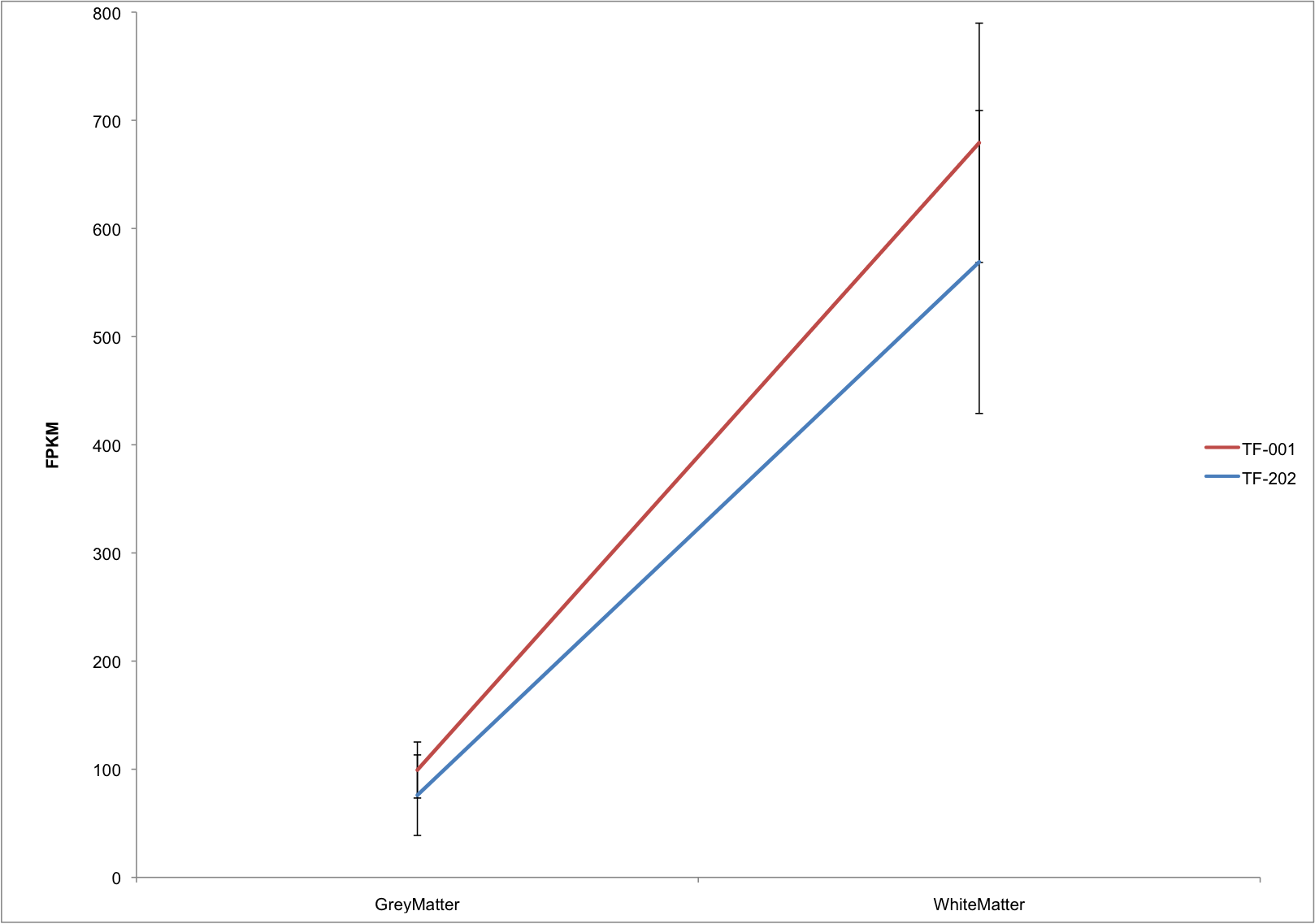

Supplement: Figure S5 — Expression levels of transferrin (TF) isoforms. There were two TF splice variants expressed across GM and WM. Both splice vairants contributed almost equal levels of expression to both GM and WM. TF-001 was upregulated 7x in WM when compared to GM. TF-202 was a novel splice varaints, it was expressed at higher levels in WM than in GM, however the changes in expression was not considered to be statistically significant. Error bars are ± standard error. (TIFF) [file pone.0078480.s009.tiff]

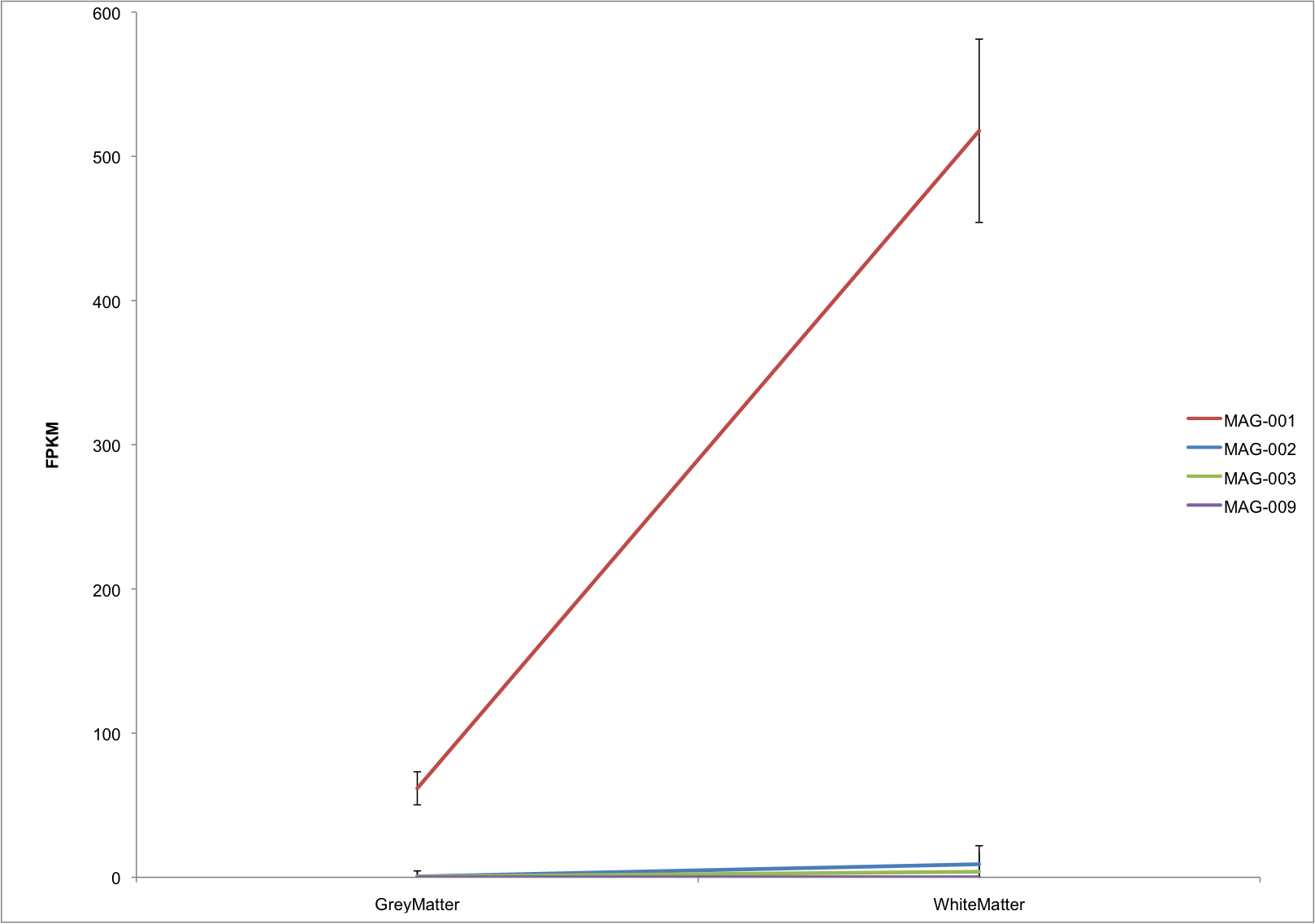

Supplement: Figure S6 — Expression levels of myelin associated glycoprotein (MAG) isoforms. There were four MAG splice variants expressed across GM and WM. MAG-001 was the dominant isoform and was up regulated 8x in WM when compared to GM. The three other splice variants (MAG-002, MAG-003, MAG-009) were expressed at low levels in both GM and WM. MAG-009 was a novel splice variant. Error bars are ± standard error. (TIFF) [file pone.0078480.s010.tiff]

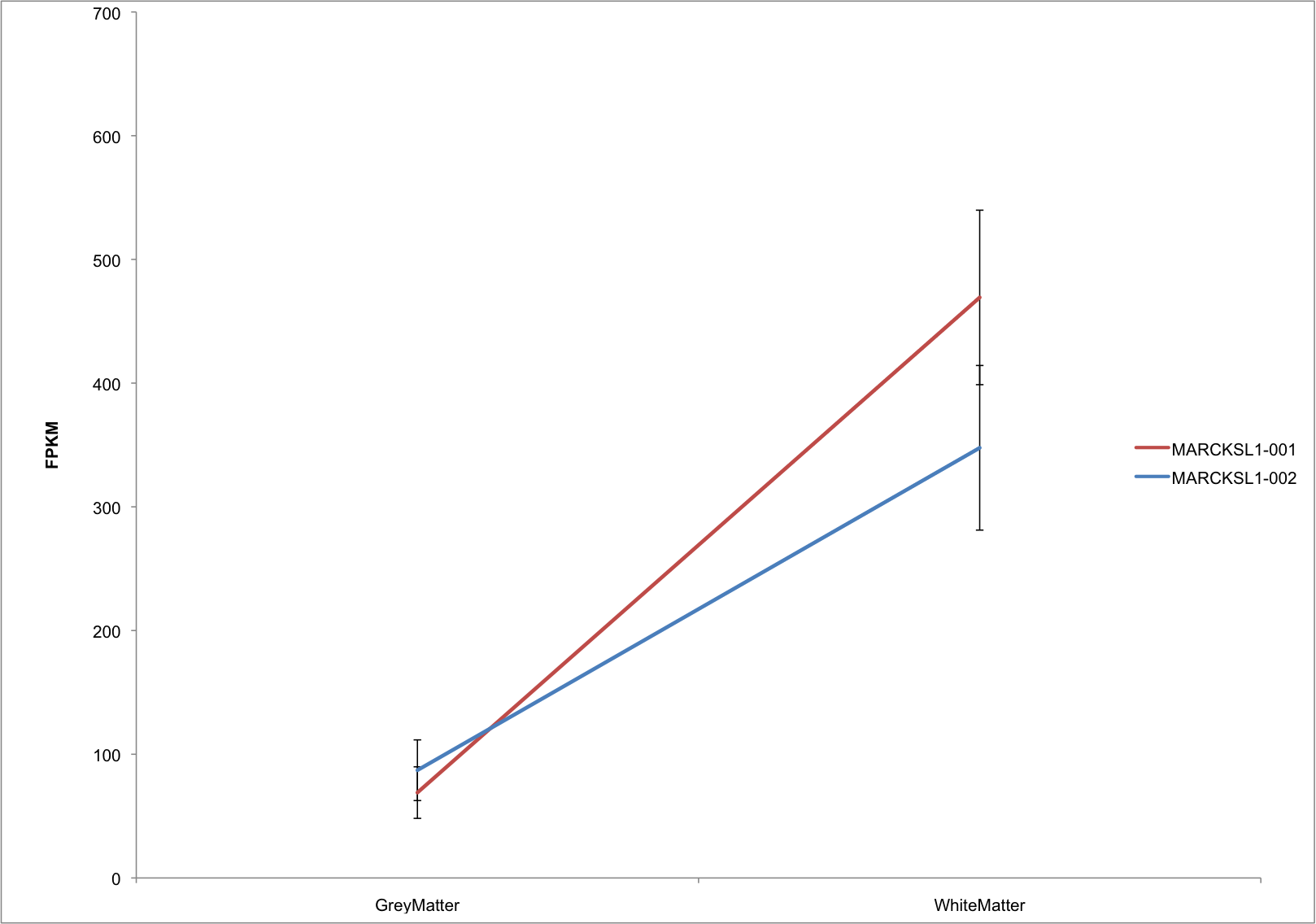

Supplement: Figure S7 — Expression levels of MARCKS-like 1 (MARCKSL1) isoforms. There were two MARCKSL1 splice variants expressed across GM and WM. Both splice vairants contributed high levels of expression to both GM and WM. MARKSL1-001 was upregulated 7x in WM when compared to GM. MARCKSL1-002 was a novel splice varaints, it was also expressed at higher levels in WM than in GM, however the changes in expression was not considered to be statistically significant. Error bars are ± standard error. (TIFF) [file pone.0078480.s011.tiff]

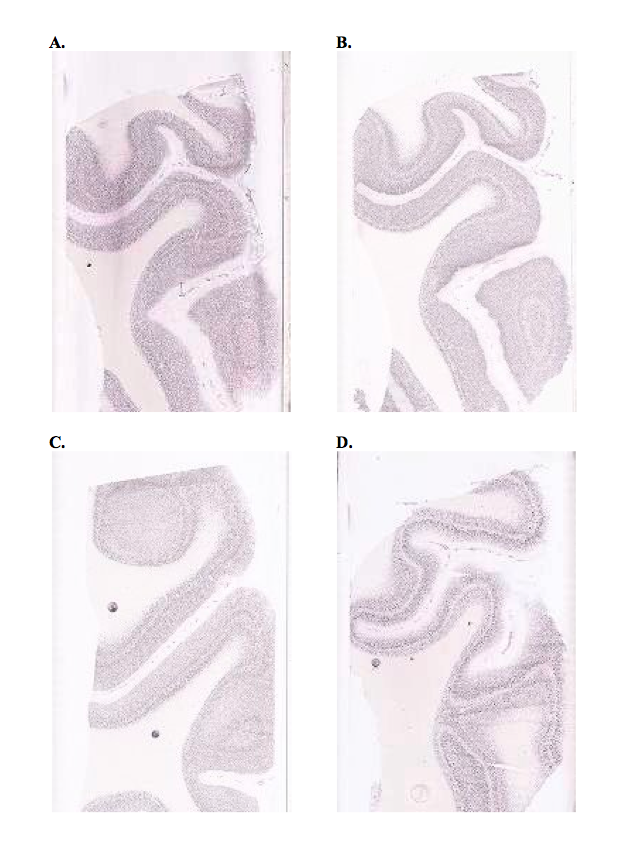

Supplement: Figure S8 — Allen Human Brain Atlas insitu hybridisation for the RGS4, CAMK2A, SLC17A7 and NEFM genes. A. RGS4: Regulator of G-protein signalling 4 GM FPKM: 100.75 WM FPKM: 1.52. Slide from the dorsolateral cortex of a healthy 20-year-old male. The slide shows high levels of expression in GM. B. CAMK2A: Calcium/calmodulin-dependent protein kinase II alpha GM FPKM: 233.16 WM FPKM: 5.27. Slide from the dorsolateral cortex of a healthy 20-year-old male. The slide shows high levels of expression in GM. C. SLC17A7: Solute carrier family 17 (sodium-dependent inorganic phosphate cotransporter), member 7 GM FPKM: 270.79 WM FPKM: 7.39. Slide from the dorsolateral cortex of a healthy 20-year-old male. The slide shows high levels of expression in GM. D. NEFM: Neurofilament, medium polypeptide GM FPKM: 224.01 WM FPKM: 4.09. Slide from the dorsolateral cortex of a healthy 20-year-old male. The slide shows high levels of expression in GM. Source: Allen Human Brain Atlas (Hawrylycz et al. 2012 and ©2012 Allen Institute for Brain Science. Allen Human Brain Atlas [Internet]. Available from: http://human.brain-map.org/). (TIFF) [file pone.0078480.s012.tiff]
